# Harnessing Plant-Based Nanoparticles for Targeted Therapy: A Green Approach to Cancer and Bacterial Infections

**DOI:** 10.3390/ijms26147022

**Published:** 2025-07-21

**Authors:** Mirela Claudia Rîmbu, Daniel Cord, Mihaela Savin, Alexandru Grigoroiu, Mirela Antonela Mihăilă, Mona Luciana Gălățanu, Viorel Ordeanu, Mariana Panțuroiu, Vasilica Țucureanu, Iuliana Mihalache, Oana Brîncoveanu, Adina Boldeiu, Veronica Anăstăsoaie, Carmen Elisabeta Manea, Roxana-Colette Sandulovici, Marinela Chirilă, Adina Turcu-Știolică, Emilia Amzoiu, Victor-Eduard Peteu, Cristiana Tănase, Bogdan Firtat, Carmen-Marinela Mihăilescu

**Affiliations:** 1 Pharmacy Faculty, “Titu Maiorescu” University, 040314 Bucharest, Romania; mirela.rimbu@prof.utm.ro (M.C.R.); mirela.mihaila@prof.utm.ro (M.A.M.); luciana.galatanu@prof.utm.ro (M.L.G.); ordeanu_viorel@yahoo.com (V.O.); carmen.manea@nipne.ro (C.E.M.); roxana.sandulovici@prof.utm.ro (R.-C.S.); marinela.chirila@prof.utm.ro (M.C.); 2 Medical Doctoral School, Titu Maiorescu University, 040317 Bucharest, Romania; 3 National Institute for Research and Development in Microtechnologies (IMT Bucharest), 072996 Bucharest, Romania; mihaela.savin@imt.ro (M.S.); alexandru.grigoroiu@imt.ro (A.G.); vasilica.tucureanu@imt.ro (V.Ț.); iuliana.mihalache@imt.ro (I.M.); oana.brincoveanu24@gmail.com (O.B.); adina.boldeiu@imt.ro (A.B.); veronica.anastasoaie@gmail.com (V.A.); bogdan.firtat@imt.ro (B.F.); 4 Stefan S. Nicolau Institute of Virology, 030304 Bucharest, Romania; 5 Horia Hulubei National Institute for R&D in Physics and Nuclear Engineering, 30 Reactorului Street, 077125 Magurele, Romania; 6 Pharmacoeconomics Department, University of Medicine and Pharmacy of Craiova, 200349 Craiova, Romania; adina.turcu@umfcv.ro; 7 Department of Physical Chemistry, University of Medicine and Pharmacy of Craiova, 200349 Craiova, Romania; emanro2002@yahoo.com; 8 Ultrastructural Pathology and Bioimaging Laboratory, Institute of Pathology Victor Babeș, Splaiul Independentei 99-101, 050096 Bucharest, Romania; peteuvictoreduard@gmail.com; 9 Faculty of Chemical Engineering and Biotechnologies, Politehnica University of Bucharest, 011061 Bucharest, Romania; 10 Victor Babes National Institute of Pathology, 050096 Bucharest, Romania; cristianatp@yahoo.com; 11 Department of Cell Biology and Clinical Biochemistry, Titu Maiorescu University, 031593 Bucharest, Romania; 12 Doctoral School, University of Medicine and Pharmacy of Craiova, 200349 Craiova, Romania

**Keywords:** green nanoparticles, antitumoral, biosynthesis, nanomedicine, antimicrobial, nanoformulation, apoptosis

## Abstract

This study investigates the antioxidant, antimicrobial, and antitumor activities of *Taraxacum officinale* (Dandelion) and *Artemisia annua* (Sweet Wormwood) extracts, along with their role in the green synthesis of gold (AuNPs) and silver nanoparticles (AgNPs). Bioreduction was achieved using aqueous and ethanolic extracts (100 mg/mL), enabling solvent-dependent comparisons. Nanoparticles were characterized using ultraviolet–visible spectroscopy (UV–Vis), fluorescence spectroscopy, scanning electron microscopy (SEM), dynamic light scattering (DLS), high-resolution transmission electron microscopy (HRTEM), and zeta potential analysis. Each technique revealed complementary aspects of nanoparticle morphology, size, and stability, with UV–Vis indicating aggregation states and DLS confirming solvent-related size variation even at 3–5% ethanol. Gold nanoparticles synthesized from Dandelion showed strong antibacterial activity against *Staphylococcus aureus*, while silver nanoparticles from both plants were effective against *Escherichia coli*. Cytotoxicity assays indicated that silver nanoparticles obtained from ethanolic Dandelion extract containing 3% ethanol in aqueous solution (AgNPsE_ETOH3%_-D) significantly reduced LoVo (*p* = 4.58 × 10^−3^) and MDA-MB-231 (*p* = 7.20 × 10^−5^) cell viability, with high selectivity indices (SI), suggesting low toxicity toward normal cells. Gold nanoparticles synthesized from aqueous Dandelion extract (AuNPsEaq-D) also showed favorable SI values (2.16 for LoVo and 8.41 for MDA-MB-231). Although some formulations demonstrated lower selectivity (SI < 1.5), the findings support the therapeutic potential of these biogenic nanoparticles. Further in vivo studies and pharmacokinetic evaluations are required to validate their clinical applicability.

## 1. Introduction

Plants and their natural compounds have been utilized in traditional medicine for centuries and remain a primary source for novel drug development, with many undergoing clinical trials to establish their therapeutic potential. The key constituents responsible for their pharmacological effects are secondary metabolites, including polyphenols (flavonoids, anthocyanidins, quinones, tannins, coumarins, phenolic acids), terpenes, alkaloids, lectins, polycarbohydrates, and polypeptides [1]. Among these, polyphenols are of particular interest due to their broad range of pharmacological activities, including antioxidant, anti-inflammatory, antibacterial, antiviral, antifungal, and anticancer effects [2,3]. Moreover, flavonoids—the most bioactive phenolic compounds—modulate key cellular and molecular mechanisms involved in inflammation, carcinogenesis, and microbial survival, making them promising candidates for phytotherapy and nanomedicine applications.

Two such examples are *Taraxacum officinale* (Dandelion) and *Artemisia annua* (Sweet Wormwood)*,* widely dispersed and fast-growing plants from the *Asteraceae* family, found in Romania’s wild flora. They have been extensively studied for their medicinal properties, particularly due to their high content of bioactive compounds with antioxidant, antimicrobial, and anticancer activities. Dandelion contains bioactive compounds, such as sesquiterpenes, phenolics, terpenoids, sterols, and flavonoids, which contribute to its pharmacological properties [4]. Sesquiterpenoids, such as taraxinic acid, contribute to both its anti-inflammatory and antibacterial properties. Flavonoids, including apigenin, luteolin, and quercetin, along with flavanol glycosides (e.g., luteolin-7-β-D-glucopyranose, quercetin-7-β-D-glucopyranoside), are responsible for anti-inflammatory and antioxidant effects [5,6]. Meanwhile, sterols, such as taraxasterol, β-sitosterol, β-sitosterol-3-O-β-D-glucoside, gigantursenol A, along with novel triterpenoids (lupane, euphane, and bauerane), have been successfully isolated from Dandelion roots, demonstrating antitumor, antioxidant, and antibacterial effects [7]. Additionally, phenolic compounds from Dandelion leaves and flowers, including hydroxycinnamic acid esters (chlorogenic acid, chicoric acid, and mono-caffeoyl tartaric acid), have been recognized for their antidiabetic properties [8,9]. One of the most significant bioactive components of Dandelion is Dandelion polysaccharide (DP), which has been reported to exhibit strong antioxidant, antibacterial, anti-inflammatory, and anticancer properties [4]. Recent studies suggest that DP’s physicochemical properties and structural configuration influence its anticancer activity, particularly against hepatocellular carcinoma (HepG2) cells [10,11].

Similarly, Sweet Wormwood exhibits numerous therapeutic effects, including antihyperlipidemic, antiplasmodial (malaria treatment), anticonvulsant, anti-inflammatory, antimicrobial, anticholesterolemic, antiviral, antioxidant, antitumor, and anti-obesity activities [12,13]. Extracts from Sweet Wormwood demonstrated promising antitumor effects by inhibiting human gastric adenocarcinoma (AGS) cell-line proliferation, reducing tumor growth, and inducing apoptosis in MDA-MB-231 breast cancer cells [14,15]. Recent studies have leveraged the bioactive compounds in Dandelion and Sweet Wormwood for the green synthesis of metallic nanoparticles, offering enhanced antioxidant, antibacterial, antifungal, and antitumor properties. These nanoparticles have been reported to exhibit enhanced antioxidant, antibacterial, antifungal, and antitumor properties, making them promising candidates for nanomedicine applications [16,17,18].

Recent advances in nanotechnology have further demonstrated the potential of plant-mediated synthesis of metallic nanoparticles for biomedical applications. Montazersaheb et al. (2024) [19] synthesized silver nanoparticles (AgNPs) using pumpkin peel extract and demonstrated their potential as radiosensitizers against triple-negative breast cancer (TNBC) cells. These AgNPs enhanced the efficacy of radiotherapy by inducing apoptosis through pathways involving Bax (Bcl-2-associated X protein), p53 (tumor protein p53), and the PERK/CHOP axis (protein kinase RNA-like endoplasmic reticulum kinase/C/EBP homologous protein axis) [19]. Similarly, İpek et al. (2024) [20] utilized *Allium cepa* L. (onion) peel aqueous extract for the green synthesis of gold nanoparticles (AuNPs). The resulting AuNPs exhibited significant antipathogenic, antioxidant, and anticholinesterase activities, highlighting the therapeutic potential of plant-mediated nanoparticle synthesis [20,21]. Building on these findings, green-synthesized nanoparticles have shown potent antitumoral properties by inducing apoptosis and inhibiting tumor proliferation [22]. Their effectiveness extends beyond anticancer applications, as comparative studies with conventional chemotherapeutic agents have revealed their potential to enhance drug efficacy, while minimizing adverse side effects [23]. Moreover, their broad-spectrum antibacterial activity has been extensively studied, demonstrating significant inhibition of multi-drug-resistant bacteria [24,25]. These findings underscore the therapeutic versatility of green-synthesized nanoparticles, reinforcing their potential as an eco-friendly and efficient alternative to conventional treatments in oncology and antimicrobial therapies. These studies underscore the efficacy of green-synthesized nanoparticles in cancer treatment and antimicrobial applications, providing a foundation for our research on Dandelion and Sweet Wormwood extracts in nanoparticle synthesis.

The use of green-synthesized nanoparticles (NPs) in biomedical applications is gaining increasing interest due to their biocompatibility, tunable physicochemical properties, and improved therapeutic efficacy. Additionally, their antioxidant and immunomodulatory properties further support their use in therapeutic applications [26]. Compared to chemically synthesized nanoparticles, green-synthesized NPs offer significant advantages, including reduced toxicity, environmentally friendly production, and improved biological interactions [27]. Several studies have demonstrated the powerful reducing capacity of plant polyphenols in forming metallic nanoparticles [17,28,29]. However, most studies have not quantitatively assessed the presence of phytotherapeutic compounds in the final colloidal solution [30,31,32,33,34,35]. This lack of data hinders their ability to correlate bioactive compound concentrations with nanoparticle stability and therapeutic effects, limiting reproducibility and standardization. To address this gap, our study systematically quantifies the residual phytotherapeutic compounds after nanoparticle synthesis using dosage methods, Fourier transform infrared spectroscopy (FTIR), fluorescence analysis, and ultraviolet–visible spectroscopy (UV-Vis). This approach ensures a more accurate understanding of how bioactive compounds influence nanoparticle properties and biological activity, contributing to the optimization of green synthesis for biomedical applications.

The aim of this study is to investigate the therapeutic potential of bioactive compounds derived from Sweet Wormwood (*Artemisia annua*) and Dandelion (*Taraxacum officinale*), sourced from Romania’s wild flora, and to evaluate their impact on the stability, size, and biological activity of green-synthesized nanoparticles. This research explores the eco-friendly synthesis of silver (Ag) and gold (Au) nanoparticles using aqueous and alcoholic extracts (50:50 and 50:30 water-to-ethanol ratios) from these plants. The physicochemical and biological properties of the synthesized nanoparticles were systematically characterized using scanning electron microscopy (SEM), high-resolution transmission electron microscopy (HTEM), FTIR, fluorescence analysis, UV-Vis spectroscopy, dynamic light scattering (DLS), and polydispersity index (PDI) measurements. Additionally, biological assessments, including the DPPH assay (2,2-diphenyl-1-picrylhydrazyl assay), agar diffusion method, and MTS cytotoxicity assay (3-(4,5-dimethylthiazol-2-yl)-5-(3-carboxymethoxyphenyl)-2-(4-sulfophenyl)-2H-tetrazolium assay), were performed. The antitumoral activity of the nanoparticles was evaluated against MDA-MB-231 (human breast adenocarcinoma), LoVo (human colon adenocarcinoma), and HepG2 (human liver hepatocellular carcinoma) cancer cell lines. To assess their potential for safer therapeutic applications, the selectivity index (SI) was determined as a measure of lower toxicity compared to the reference chemotherapeutic drugs, DOX (doxorubicin) and CisPt (cisplatin).

## 2. Results

### 2.1. UV-VIS Spectra Characterization

The UV-VIS spectrophotometric analysis confirmed the formation of silver and gold nanoparticles reduced by Dandelion and Sweet Wormwood extracts.

**Figure 1:**
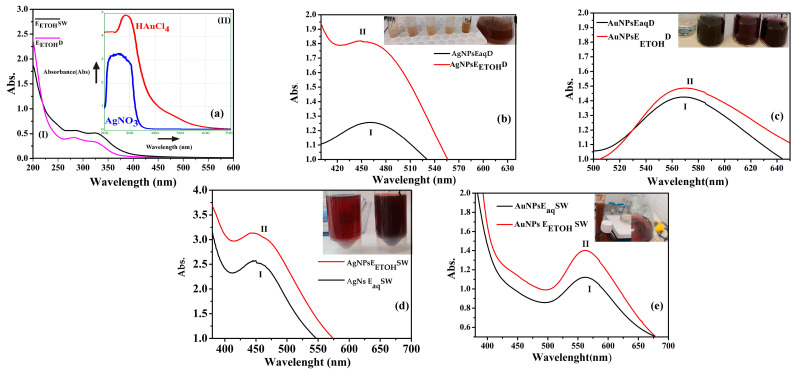
(**a**) UV–Vis spectra of (I) *Artemisia annua* (sweet wormwood, black line) and *Taraxacum officinale* (dandelion, magenta line) ethanol extracts, and (II) 1 mM AgNO_3_ (blue line) and 0.5 mM HAuCl_4_·3H_2_O (red line); (**b**) UV–Vis spectra of silver nanoparticles synthesized with dandelion extract in aqueous solution (AgNPsEaq-D, I—black line) and in ethanol (AgNPsE_ETOH_-D, II—red line). Inset: color change from pale yellow to brown during AgNPs formation; (**c**) UV–Vis spectra of gold nanoparticles synthesized with dandelion extract in aqueous solution (AuNPsEaq-D, I—black line) and in ethanol (AuNPsE_ETOH_-D, II—red line). Inset: color change to violet during AuNPs formation; (**d**) UV–Vis spectra of silver nanoparticles synthesized with sweet wormwood extract in aqueous solution (AgNPsEaq-SW, I—black line) and in ethanol (AgNPsE_ETOH_-SW, II—red line). Inset: color change to brown during AgNPs formation; (**e**) UV–Vis spectra of gold nanoparticles synthesized with sweet wormwood extract in aqueous solution (AuNPsEaq-SW, I—black line) and in ethanol (AuNPsE_ETOH_-SW, II—red line). Inset: color change to red-violet during AuNPs formation. Inset: color change to red-violet during AuNPs formation.

The absorption spectra of the extracts, silver nitrate, and chloroauric acid are presented in Figure 1a(II).

Figure 1b–e shows the UV-VIS spectra obtained for nanoparticle solutions formed via bioreduction using either aqueous or alcoholic plant extracts.

In Figure 1b, the UV-VIS spectrum of AgNP_S_Eaq-D (curve I) is compared with that of AgNP_S_E_ETOH_-D (curve II), indicating differences resulting from the use of aqueous versus alcoholic Dandelion extracts.

Figure 1c presents the spectra of AuNP_S_Eaq-D (I) and AuNP_S_E_ETOH_-D (II), revealing spectral shifts between nanoparticles synthesized with aqueous and alcoholic Dandelion extracts.

Similarly, Figure 1d shows the spectra for AgNP_S_Eaq-SW (I) and AgNP_S_E_ETOH_-SW (II), corresponding to silver nanoparticles reduced with Wormwood aqueous and alcoholic extracts.

For gold nanoparticles reduced with Wormwood extracts, the corresponding spectra are shown in Figure 1e—AuNP_S_Eaq-SW (I) for the aqueous extract and AuNP_S_E_ETOH_-SW (II) for the alcoholic extract.

### 2.2. Total Polyphenol Content and Total Flavonoids Content

The experimental analysis revealed that Artemisia herba extracts contained a higher amount of total polyphenols and flavonoids compared to *Taraxacum herba* extracts. Specifically, the total polyphenol content (TPC) was 16.82 ± 0.12 mg GAE/g DW in Artemisia herba, compared to 15.78 ± 0.35 mg GAE/g DW in Taraxacum herba. Similarly, the total flavonoid content (TFC) was 9.17 ± 0.73 mg RE/g DW in Artemisia herba and 7.95 ± 0.65 mg RE/g DW in Taraxacum herba. The TPC and TFC were also quantitatively determined in the colloidal nanoparticle solutions synthesized from these plant extracts. These values are summarized in Table 1.

### 2.3. Fluorescence of Nanoparticles

Fluorescence emission was observed in all nanoparticle samples. Emission bands were recorded in the range of 300–380 nm and 600–700 nm, corresponding to the presence of polyphenolic compounds and chlorophyll-related molecules, respectively.

Figure S2a–f in the Supplementary Materials displays the recorded fluorescence spectra for silver and gold nanoparticles synthesized using both aqueous and alcoholic extracts of Taraxacum officinale and Artemisia annua. The intensity and position of the emission peaks varied slightly depending on the type of extract and metal used.

### 2.4. FTIR

FTIR measurements confirmed the presence of specific functional groups involved in nanoparticle formation and stabilization.

Table S1 presents the tentative assignment of absorption bands observed for the six analyzed samples.

**Figure 2:**
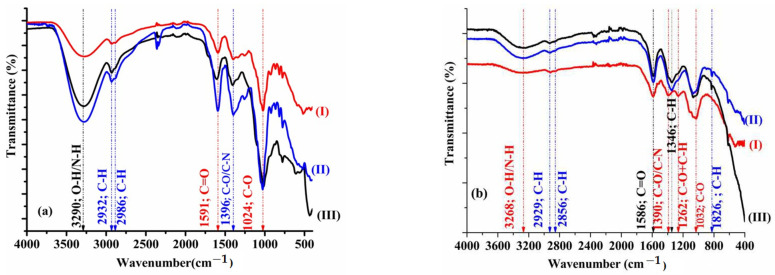
(**a**) ATR-FTIR spectra for Dandelion-based sample: (I) E_ETOH_-D; (II) AuNPsE_ETOH_-D; (III) AgNPsE_ETOH_-D; (**b**) ATR-FTIR spectra for Sweet Wormwood based sample: (I) E_ETOH_-SW; (II) AuNPsE_ETOH_-SW; (III) AgNPsE_ETOH_-SW.

Figure 2a shows the FTIR spectra for Dandelion, as follows: alcoholic extract (I), AuNPsE_ETOH_-D (II), and AgNPsE_ETOH_-D (III), revealing distinct spectral shifts after nanoparticle synthesis.

Figure 2b shows similar spectra for Sweet Wormwood, as follows: alcoholic extract (I), AuNPsE_ETOH_-SW (II), and AgNPsE_ETOH_-SW (III). Notable changes in peak positions and intensities suggest chemical interaction between phytochemicals and the metal nanoparticle surfaces.

### 2.5. DLS Analysis, PDI, Zeta Potential, SEM and TEM

Table 2 displays the PDI values, average hydrodynamic diameter, and zeta potential for all obtained nanoparticles.

The DLS spectra and the corresponding zeta potential values for all analyzed samples are presented in Supplementary Figures S3.1(a1–d2) and S3.2(a1–d2). The SEM images, illustrating the morphology of the synthesized nanoparticles, are provided in Supplementary Figure S4.

**Figure 3:**
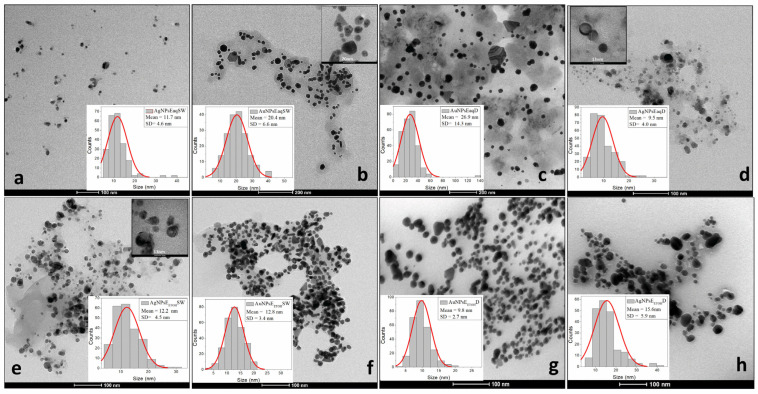
TEM for NPs (around 200 nanoparticles) aqueous extracts plant-based sample: (**a**) AgNPsEaq-SW (graph inset Mean = 11.7 nm SD = 4.6 Min = 6 max = 37); (**b**) AuNPsEaq-SW (Mean = 20.4 nm SD = 6.6 Min = 8 max = 41); (**c**) AuNPsEaq-D (Mean = 26.9 nm SD = 14.3 Min = 5 max = 133); (**d**) AgNPsEaq-D (Mean = 9.5 SD = 4.0 Min = 4 max = 25); TEM for NPs (around 200 nanoparticles) from ethanolic extracts from plant based sample: (**e**) AgNPsE_ETOH_-SW (Mean = 12.2 nm SD = 4.5 Min = 5 max = 30); (**f**) AuNPsE_ETOH_-SW; (**g**) AuNPsE_ETOH_D; (**h**) AgNPsE_ETOH_-D.

Figure 3a–h presents TEM images for all analyzed samples, and Table 3 contains the corresponding size and morphology of nanoparticles as per TEM measurements.

### 2.6. Biological Tests

#### 2.6.1. Antioxidant Proprieties

Table S3 shows the percentage of DPPH inhibition obtained by the green nanoparticles and by their corresponding extracts.

#### 2.6.2. Antimicrobial and Antifungal Proprieties

**Figure 4:**
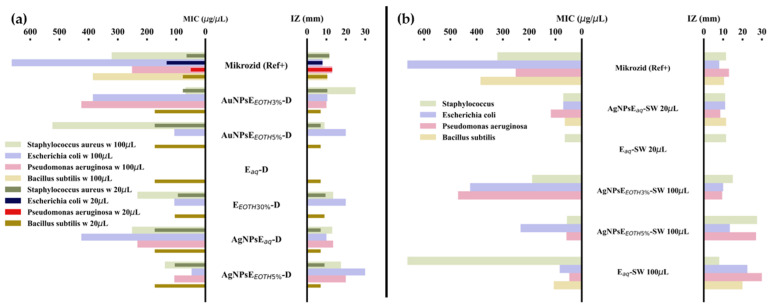
The MIC (µg/mL) and corresponding IZ (mm) for the all sample based on (**a**) Dandelion and (**b**) Sweet Wormwood.

Figure 4a,b illustrates the zone of inhibition versus inhibition concentration calculated for Dandelion and, respectively, Sweet Wormwood samples using Equation (4). The antifungal effect of the nanoparticles is presented in Supplementary Figure S5 for the Dandelion samples and Figure S6 for the Sweet Wormwood samples.

#### 2.6.3. Antitumoral Effect

- MTS Cytotoxicity Assay

**Figure 5:**
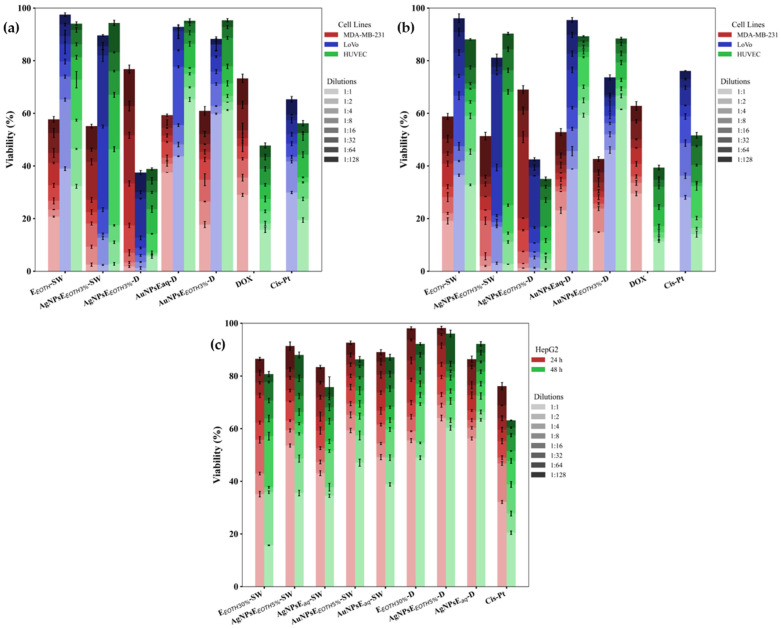
Effect of different dilutions of sample based on dandelion and sweet wormwood on the viability (%) of human cancer cell lines, as follows: breast cancer (MDA-MB-231), the human colorectal adenocarcinoma cancer cell line LoVo, and the human umbilical vein endothelial cells HUVEC at (**a**) 24 h and (**b**) 48 h; (**c**) effect of different sample dilutions based on Dandelion and Sweet Wormwood on the viability (%) of HepG2 at 24 h and 48 h. Dilutions are given by the shade of the overlapping bar plots, with a lighter shade corresponding to less dilution.

Studies were carried out in vitro to investigate the effect of the tested samples on the LoVo and MDA-MB-231 human tumor cells and liver tumor line HepG2 when compared to the HUVEC normal cell line (Figure 5a–c). The positive controls of the study were CisPt and DOX, which are commonly used to treat colon and breast cancer, respectively. To evaluate cell viability, dilutions of the initial colloidal solutions were made, and the cell lines were exposed for 24 and 48 h.

- Statistical Analysis

Table S7 (Supplementary) summarizes the statistical analysis results, highlighting the significant differences between green-synthesized nanoparticles and conventional chemotherapeutics (Cis-Pt and DOX) across various cancer and normal cell lines.

- Comparative Analysis of IC50 and Selectivity Index at 24 h and 48 h

**Figure 6:**
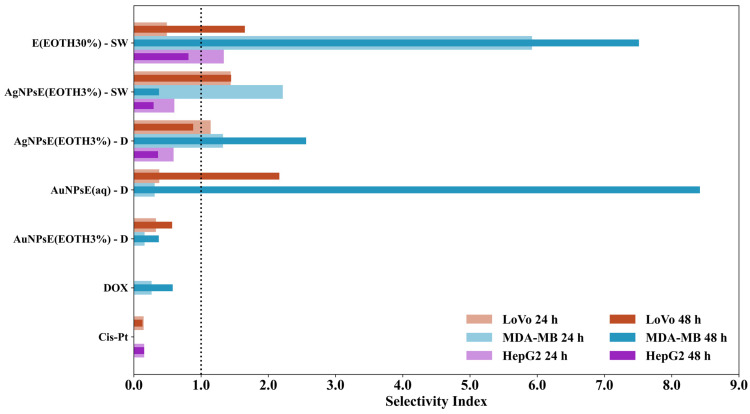
Evolution of Selectivity Index (SI) values over time for all tumor cell lines. The figure illustrates the variation in Selectivity Index (SI) across different tumor cell lines over time. SI is calculated as the ratio between IC50 in normal cells and IC50 in tumor cells, indicating the specificity of nanoparticles towards cancer cells.

Tables S8 and S9 present the SI and IC50 values after sample treatment on LoVo and MDA-MB-231 cell lines, along with their corresponding errors, at 24 and 48 h of treatment. Table S10 displays the SI and IC50 values after sample treatment on HepG2 cells. Figure 6 illustrates the evolution of the SI for all tumor cell lines over time.

## 3. Discussion

### 3.1. UV-VIS and Fluorescence

- Dandelion gold/silver nanoparticles

The successful formation of silver nanoparticles (AgNPs) and gold nanoparticles (AuNPs) using *Taraxacum officinale* extracts was confirmed by visual color changes and characteristic surface plasmon resonance (SPR) bands observed in UV–Vis spectroscopy. In the case of AgNPs, the color of the reaction mixture changed to dark brown approximately 30 min after the addition of either extract, suggesting the reduction in Ag^+^ ions to elemental silver (Ag^0^). This transformation is visible in the inset of Figure 1b, which illustrates the color shift of the silver nitrate–dandelion mixture.

UV–Vis spectra revealed distinct SPR peaks associated with the AgNPs. The ethanolic extract (AgNPs E_ETOH_-D) showed a peak at 462 nm (Figure 1b(I)), whereas the aqueous extract (AgNPs E_aq_-D) displayed a peak at 450 nm (Figure 1b(II)). The red shift observed in the ethanolic extract indicates the formation of larger particles, which is consistent with spectral estimations; AgNPs synthesized with the aqueous extract had an average size of 37.57 nm, while those synthesized with the ethanolic extract measured 73.97 nm.

These findings are in line with previous studies reporting SPR bands between 400–480 nm for silver nanoparticles synthesized using *Taraxacum officinale* flowers [36]. Furthermore, other works employing high-resolution transmission electron microscopy (HRTEM) reported particle sizes around 15 nm with a maximum SPR wavelength of 435 nm for AgNPs synthesized via aqueous Dandelion extracts, highlighting the variability depending on extract concentration, composition, and conditions [37].

The differences in particle size and SPR behavior between the two extracts are attributed to solvent polarity and biochemical composition. Ethanol, being less polar, facilitates the extraction of more hydrophobic phytochemicals, which may influence nanoparticle growth and aggregation. However, the aqueous extract exhibited a higher polyphenol content, which may enhance the nucleation rate and limit particle growth, resulting in smaller, more uniformly distributed nanoparticles.

Phytochemical analysis (Table 1) showed that both types of AgNPs contained gallic acid, while rutin was detected exclusively in AgNPs E_ETOH_-D. The presence of rutin may contribute to different stabilization or reduction dynamics, potentially explaining the observed differences in particle size and optical properties.

For gold nanoparticles, both ethanolic and aqueous extracts produced colloids with a violet coloration, as seen in the inset of Figure 1c, confirming the formation of AuNPs. The UV–Vis spectra of these samples exhibited identical SPR peaks at 568 nm, as illustrated in Figure 1c, indicating similar nanoparticle size and optical characteristics in both extract types.

Size estimations for AuNPs were calculated using the Haiss equation [38], yielding theoretical values of 109.15 nm for AuNPs E_ETOH_-D and 109.81 nm for AuNPs Eaq-D. Despite having the same λ_max, slight differences in the inflection point wavelength (λ_0_) of the UV–Vis curves were observed, suggesting minor variations in particle environment or morphology.

In summary, the formation and characteristics of AgNPs and AuNPs synthesized with *Taraxacum officinale* extracts are significantly influenced by extract type and solvent. While ethanol enriches the extract in certain biomolecules (e.g., rutin), the aqueous medium preserves higher polyphenol concentrations. These chemical differences impact the size, stability, and SPR features of the resulting nanoparticles, offering potential tunability for specific biomedical or catalytic applications.

- Sweet Wormwood gold/silver nanoparticles

The characteristic band of the silver nanoparticles between 400 and 450 nm should be present only in the solution containing silver nanoparticles. Consequently, the UV-VIS spectra for both colloidal solutions exhibited a band at 441 nm for AgNPsEaq-SW (Figure 1d(I)) and 445 nm for AgNPsE_ETOH_-SW (Figure 1d(II)). The corresponding sizes of the AgNPs, determined using the same theoretical methods as applied to those from Dandelion extracts through UV-VIS spectral analysis, were 38.48 nm for AgNP_S_Eaq-SW and 41.21 nm for AgNPsE_ETOH_-SW [38].

The bioreduction of gold from Sweet Wormwood led to the formation of a maximum absorbance at 563 nm for AuNPs Eaq-SW corresponding to a nanoparticle size of about 105.67 nm (Haiss equation), as shown in the spectra from Figure 1e(I). Figure 1e(II) shows the UV-VIS spectra of AuNPs E_ETOH_SW, which has a maximum absorbance at 557 nm and a corresponding calculated size of 111.12 nm. The inset of Figure 1e presents a color change from brown to red-violet, confirming the formation of gold nanoparticles. The time required for the reduction in metallic nanoparticles with Sweet Wormwood was 40 min; although, an increase in this maximum was observed after 24 h, indicating that nanoparticle formation continues over time. Although the extract-to-salt ratio required for bioreduction and nanoparticle formation was the same for all syntheses, a longer time was needed for the gold nanoparticles from wormwood to form, taking 40 min compared to the 30 min required for silver nanoparticles. It is possible that the reduction process for gold requires more time, because Au ions are more difficult to reduce to the zero-valent state compared to Ag ions. While the maximum absorbance remained unchanged, a narrowing of the full width at half maximum (FWHM) was observed over time, suggesting that the nanoparticles become more homogeneous in solution. However, there was a tendency for flocculation, especially in gold nanoparticles, which might indicate that the bonds between the therapeutic compounds and the nanoparticles can break over time, leading to aggregation. DLS studies, zeta potential, and polydispersity index better clarify this aspect.

- Fluorescence of nanoparticles colloidal solutions

The obtained green nanoparticles act as carriers for polyphenolic compounds. According to literature, the emission region from 300 to 380 nm corresponded to polyphenols, and the emission region from 600 to 700 nm was ascribed to chlorophyll and its derivatives [39]. For AgNPsE_ETOH_-D, the maximum excitation and emission wavelengths were at 270 nm/360 nm and 310 nm/435 nm, respectively, as indicated in Figure S2a,b. For AgNPsEaq-D, the intensity of the fluorescence band at 360 nm/480 nm was 1 × 10^5^ (Figure S2b,c), lower than Ag NPs obtained from the D ethanol extract (1.8 × 10^5^), and shifted after excitation to 270 nm. This may indicate a lower content of polyphenols bound to the silver nanoparticles obtained from the aqueous extract, taking into consideration that the fluorescence source is the polyphenols. In the case of AuNPsE_ETOH_-D, their maximum excitation and emission wavelengths were similar, as follows: 270 nm/380 nm and 400 nm/470 nm, respectively Figure S2e,f. The intensity of the fluorescence bands of AuNPs was higher compared to AgNPs, regardless of whether the extract was aqueous or ethanolic. This demonstrates a greater capacity of the green gold nanoparticles from Dandelion/Sweet Wormwood to act as carriers of polyphenols compared to the silver nanoparticles. The aqueous and ethanolic solutions with Ag/Au NPs obtained from Sweet Wormood exhibited similar behavior, which suggests that the obtained green nanoparticles act as carriers of polyphenols, potentially having significant biological and pharmaceutical importance. Characterizing the colloidal stability of these nanoparticles is crucial, especially since no chemical stabilizing agents were used. Stability tests were using DLS analysis, polydispersity index, and zeta potential.

### 3.2. FTIR

From a structural point of view, the chemical composition of Dandelion and Sweet Wormwood is influenced by genetic variability, the plant organ, and the biological stage. Dandelions are rich in vitamins, inulin, phytosterols, amino acids and minerals, sesquiterpenes, triterpenes, phytosterols, and phenolic compounds in different proportions [40]. Similarly cis-epoxyocimene, cis-chrysanthenol, thujone, bornyl or chrysanthenyl acetate, chrysanthenol, chamazulene, sabinyl, 1,8-cineole, caryophyllene, myrene, sabinene, linalool, chrysanthenyl acetate, and trans-sabinyl acetate can be isolated from Sweet Wormwood [37].

The FTIR spectrum for our Dandelion extract (Figure 2a(I)) showed a broad band due to the vibration mode of the O-H bonds of alcohols and phenols hydroxyl groups superimposed on the N-H of amine compounds (4000–3000 cm^−1^). The peaks in the range of 3000–2800 cm^−1^ can be associated with stretching vibrations of C-H, and those in the range of 1600–1000 cm^−1^ can be attributed to both the stretching and deformation modes of the CO groups (C=O conjugated to the aromatic ring (1591 cm^−1^), C-O from polyphenols (1591 cm^−1^), and C-OH from alcohols or esters (1024 cm^−1^) [10,36,37]. Comparing the spectra of the metallic nanoparticles (Figure 2a(II,III)) with the spectrum of the Dandelion extract, a slight shift of the bands in the spectral domains of 4000–3000 cm^−1^ and 1600–1000 cm^−1^ is observed, suggesting the weakening of the intermolecular H-bonding and the involvement of CO groups in the anchoring of the biomolecules’ surface of metal nanoparticles [41].

Figure 2b shows the spectrum of our Sweet Wormwood extract (Figure 2b(I)) in which bands that can be associated with (i) the vibrational mode of the O-H bonds from alcohols and phenols hydroxyl groups superimposed on N-H from amine compounds (3270 cm^−1^), (ii) stretching and deformation mode of CH bonds from fatty acids or methoxy compounds (3000–2800 cm^−1^) and, respectively, cyclohexane rings (897 cm^−1^) or aliphatic groups (700–600 cm^−1^); (iii) C=O conjugated to the aromatic ring from flavonoids (1700–1500 cm^−1^); and (iv) the stretching and deformation mode of C-O bonds from alcohols or phenol compounds, cyclic, or aliphatic substituted ethers (700–600 cm^−1^) [42,43,44,45] can be seen. Comparing the spectra of the metallic nanoparticles (Figure 2b(II,III)) with the spectrum of the Wormwood extract, small shifts of the spectral bands are observed concomitant with the change in the shape of the bands below 600 cm^−1^, thus confirming the reduction in the metal ions, Mn^+^ to M^0^ (M = Au, Ag), but also their coverage with biomolecules. The anchoring of biomolecules is supported by the existence of characteristic groups of the extract in the range of 4000–600 cm^−1^. The displacement of the observed peak for the extract from 3268 cm^−1^—to 3276 cm^−1^ for Au nanoparticles and to 3243 cm^−1^ for AgNPs, concomitant with the disappearance of the bands centered at 1390 cm^−1^ and 1262 cm^−1^, in the spectrum of the SW extract, and the appearance of a band at 1346 cm^−1^ in the NPs spectra—confirm the involvement of hydroxyl and carbonyl groups in the reduction in salts and the stabilization of metal nanoparticles [41].

Consequently, the presence of polyphenolic acids and flavonoids was confirmed through FTIR analysis. These compounds tend to be more concentrated in the colloidal solutions derived from Wormwood extracts compared to those obtained from Dandelion extracts, as illustrated previously in Table 1. Considering that the phytoactive compounds identified in the colloidal solutions play dual roles as reducing and stabilizing agents, the higher content of polyphenols and flavonoids in *Artemisia annua* extracts could explain the smaller size of the resulting nanoparticles compared to those synthesized with *Taraxacum officinale*. This hypothesis is supported by the SEM and TEM analyses.

### 3.3. Comparative Analysis of Polyphenol Content

The polyphenol and flavonoid profiles identified in this study are broadly consistent with previously published findings. For instance, Epure et al. [46] reported lower TPC and TFC in *Taraxacum* tinctures (13.15 mg GAE/g DW and 6.87 mg RE/g DW, respectively), whereas Ivanov [47] found significantly higher phenolic levels (35.03 mg GAE/g DW) in ethanolic extracts of dandelion leaves. In the case of *Artemisia* species, Guo et al. [48] reported TPC and TFC values (39.58 mg GAE/g DW and 7.04 mg RE/g DW) in *A. annua*, comparable to the results observed here for *Artemisia herba*.

Moreover, broader variability was observed in other *Artemisia* species; Saunoriute et al. [49] recorded phenolic contents ranging from 176.7 to 288.59 mg RE/g DW in *A. stelleriana* and from 297.37 to 9.18 mg RE/g DW in Sweet Wormwood samples, highlighting the influence of species and extraction methods. Carvalho et al. [50] also noted a wide range of TPC values (0.22 ± 0.002 to 0.39 ± 0.000 mg GAE/g DW) in methanolic extracts of various *Artemisia* species.

Taken together, these comparisons confirm that both *Artemisia herba* and *Taraxacum herba* are valuable sources of polyphenolic and flavonoid compounds. However, the consistently higher levels found in *Artemisia* extracts in this study may account for its enhanced ability to reduce and stabilize metal ions during green nanoparticle synthesis. This likely influences key nanoparticle features, such as yield, morphology, and stability.

Notably, the elevated TPC and TFC values observed in the nanoparticle suspensions—particularly in AuNPsE_ETOH_-SW (152.15 ± 18.25 mg RE/g DW)—suggest that a substantial proportion of bioactive compounds remain associated with the nanoparticle surface post-synthesis. This retained organic corona enhances colloidal stability and may improve the biological performance of the nanomaterials. These findings are in line with previous studies indicating that polyphenol-rich plant-derived nanoparticles exhibit enhanced antimicrobial and antioxidant activities due to surface-bound phytochemicals.

### 3.4. Correlations Between Physicochemical Properties, Antioxidant Activity, Morphology, and Antimicrobial Effects

The interrelation between nanoparticle morphology, antioxidant properties, physicochemical characteristics, and antimicrobial effects provides essential insights into their biomedical potential. Our study highlights significant variations based on plant sources, solvent type, and nanoparticle composition.

#### 3.4.1. Influence of Polyphenolic Content on Nanoparticle Stability, Antioxidant Activity, and Antibacterial Action

The total polyphenol (TPC) and flavonoid (TFC) content of extracts and nanoparticles (Table 1) directly impact their stability, antioxidant activity, and antimicrobial potential. Dandelion-derived nanoparticles (D-NPs) had lower flavonoid retention, which may explain their slightly lower antioxidant performance and weaker antibacterial effects as shown in Figure 4a. Sweet Wormwood-derived nanoparticles (SW-NPs) exhibited the highest TPC and TFC values, particularly AuNPsE_ETOH_-SW (152.15 µg RE/mL), which correlates with their enhanced colloidal stability (Zeta potential: −53.29 mV, Table 2) and strong antibacterial action against *Staphylococcus aureus* (Figure 4b).

The antioxidant assay (DPPH, Table S3) revealed that AgNPsEaq-SW had the highest antioxidant activity (83.10%), correlating with their high TPC (97.90 µg GAE/mL) and a small, uniform size (11.7 ± 4.6 nm, Table 3). This formulation also exhibited the highest antibacterial effect, particularly against *Escherichia coli* and *Staphylococcus aureus*, as shown in Figure 4b. AgNPsE_ETOH_-SW and AuNPsE_ETOH_-SW showed moderate antioxidant properties (~15–16%) and moderate antimicrobial action, likely due to larger particle sizes and aggregation tendencies. AgNPs from Dandelion (AgNPsEaq-D, AgNPsE_ETOH_-D) demonstrated lower antioxidant activity (6–13%), potentially due to a lower retention of flavonoids, correlating with their weaker antimicrobial effects (Figure 4a).

#### 3.4.2. Homogeneity, Hydrodynamic Diameter, Zeta Potential, and Shape of Nanoparticles

The presence of polyphenols and flavones in Sweet Wormwood extracts positively impacted the homogeneous formation of nanoparticles and hydrodynamic diameters. Similarly to the gold nanoparticles derived from Dandelion, the average hydrodynamic diameter for gold NPs from Sweet Wormwood was larger than that seen in SEM measurements. Gold nanoparticles from both plants contained more phytotherapeutic compounds than silver ones, as confirmed by their increased fluorescence intensity (Table 1). This suggests a high rutin/polyphenol concentration, contributing to nanoparticle stabilization.

In the absence of nanoparticle purification prior to DLS analysis, high-molecular-weight compounds may encapsulate nanoparticles, increasing their hydrodynamic distribution. Polysaccharides in Dandelion extracts, known for their antitumor properties, may also contribute to nanoparticle stabilization [4,5]. The interaction of these compounds with the nanoparticle surface explains the increased hydrodynamic diameter.

Zeta potential analysis (Table 2) revealed that low zeta potential values (<+30 mV) in some formulations led to flocculation. Moreover, AgNPsE_ETOH_-D had a highly negative zeta potential (−125.82 mV), ensuring strong colloidal stability. Dandelion nanoparticles from aqueous extracts exhibited weaker stability due to insufficient electrostatic repulsion. All nanoparticles had negative charges, suggesting they were surrounded by negatively charged ionic groups, mainly polyphenols, flavones, and phenolic acids. Among Sweet Wormwood-derived nanoparticles, AgNPsEaq-SW exhibited superior stability, while Dandelion nanoparticles (except AgNPsE_ETOH_-D) were more prone to flocculation. The differences in hydrodynamic size and PDI indicate varying encapsulation thickness depending on the concentration and type of phytotherapeutic compounds. Importantly, nanoparticles synthesized using *Artemisia annua* and *Taraxacum officinale* extracts maintained their colloidal stability even five months post-synthesis. Measurements of hydrodynamic diameter and zeta potential after this period showed minimal variation, confirming their good long-term stability. Throughout this time, samples were stored at 4 °C.

Electron microscopy (TEM/SEM), as shown in Figure 3a–h (TEM) and Figure S4a–g (SEM-Supplementary Materials), confirmed the presence of triangular gold nanoparticles in both plant extracts, with sizes ranging from 20 to 35 nm (Table 3). In addition, rod-like AuNP structures were observed in Sweet Wormwood, with dimensions of 8–15 nm in width and 20–35 nm in length. The larger hydrodynamic diameters observed in the DLS measurements were attributed to hydration layers, which may influence bioactivity. SEM images further revealed that the nanoparticles were surrounded by a layer of plant extract, which helped prevent coalescence and affected the apparent size measurements.

#### 3.4.3. Physicochemical Correlations with Antioxidant and Antimicrobial Properties

- Antioxidant Activity

Green nanoparticles enhanced DPPH inhibition due to phytocompound adsorption on their surface, facilitating hydrogen donation to free radicals. This was most evident in AgNPsEaq-SW (83.10%), indicating a high antioxidant potential (Table S3). Gold nanoparticles from Sweet Wormwood retained significant polyphenol concentrations but exhibited lower antioxidant activity than AgNPs. This suggests that silver nanoparticles interact more effectively with ROS, enhancing free radical scavenging.

- Antimicrobial Activity

Dandelion-derived AgNPsE_ETOH_-D showed significant antibacterial effects against *Escherichia coli*, correlating with its highly negative zeta potential (−125 mV). SEM images confirmed large nanoparticles encapsulated in extract layers, contributing to enhanced bacterial interaction (Figure 4a). Gold nanoparticles (AuNPsE_ETOH_-D) also showed antibacterial properties, though weaker than silver. Lower AgNP concentrations generate ROS, disrupting bacterial membranes more effectively than gold. Sweet Wormwood-derived AuNPsE_ETOH_-D displayed strong activity against *Staphylococcus aureus* (Figure 4b). Antifungal activity was generally lower (Figures S5 and S6), likely due to structural differences between bacterial and fungal cell walls. Mechanisms influencing antimicrobial effects include 1. ROS production disrupting bacterial DNA and proteins [51]; nanoparticle size and shape affecting bacterial uptake [52]; biofilm disruption and metabolic interference [53]; silver ions (Ag^+^) binding to bacterial thiol groups, altering enzymatic functions [54]. The flocculation tendency of D-NPs increased bacterial wall penetration, enhancing antimicrobial activity compared to fungal activity. Additionally, DLS spectra of AgNPsE_ETOH_-D (Figure S3.2(b1)) revealed two hydrodynamic populations, suggesting an aggregation effect impacting bacterial interactions.

The Supplementary Materials contains all values for the antimicrobial activity of the tested samples in Tables S2–S6. Furthermore, recent pharmacological studies have demonstrated the potent anti-inflammatory and anticancer properties of rutin, which was also identified in most of our samples, especially those derived from Sweet Wormwood [55].

### 3.5. Implications for Biomedical Applications

The combined influence of particle size, morphology, stability, and fluorescence properties suggests distinct biomedical applications. AgNPsEaq-SW exhibited the highest antioxidant and antibacterial activity, making them strong candidates for antimicrobial coatings and wound treatments [44,56]. Additionally, triangular AuNPsEaq-D and AuNPsE_ETOH_-D demonstrated enhanced cellular uptake, which supports their potential applications in anticancer therapies and selective antibacterial treatments [43,57]. Gold nanoparticles derived from Dandelion and Sweet Wormwood extracts exhibited increased fluorescence intensity, likely due to higher phytochemical capping with rutin and polyphenols (Table 1). This fluorescence-based stability enhancement suggests superior interaction with biological membranes, making these nanoparticles promising for targeted drug delivery applications (Figure S2a–f)). Additionally, the fluorescence signature of AuNPs correlates with their ability to retain bioactive compounds, which could improve their anticancer efficacy through enhanced cellular uptake mechanisms [52]. Moreover, Dandelion-derived nanoparticles, despite their lower antioxidant activity, exhibited notable antibacterial effects, indicating alternative therapeutic applications, particularly in microbial control and biocompatible formulations. The correlation between fluorescence intensity, nanoparticle stability, and bioactivity aligns with previous studies that emphasize the role of phytochemical-induced fluorescence in nanoparticle stabilization and therapeutic action [42]. The strong fluorescence signals observed in Figure S2e,f confirm the presence of polyphenols and flavonoids surrounding AuNPs, supporting their enhanced biocompatibility and bioactivity. Additionally, Figure S2a–f illustrate how fluorescence intensity variations reflect differences in nanoparticle functionalization, further reinforcing their potential biomedical applications.

### 3.6. Therapeutic Potential and Statistical Validation of Nanoparticles in Cancer Treatment

#### 3.6.1. Cytotoxic Analysis

CisPt and DOX appear to impact the investigated tumor lines; however, their effects also extended to the normal cell line after 24 h (Figure 5a). Meanwhile, after 24 h, the ethanolic extract of Sweet Wormwood reduced the viability of breast tumor cells, an effect not evident in the other cell lines. In the case of AgNPsE_ETOH_-D and AgNPsE_ETOH_-SW (with a final colloidal solution containing 3% ethanol), there was a slight reduction in cell viability compared to treatment with the extract alone. AgNPs synthesized from Dandelion extract exhibited a significant antitumor effect on colon cancer cells (LoVo) after 24 h (χ^2^ = 58.428; *p* = 2.56 × 10^−11^) and 48 h, with a greater impact than cisplatin (CisPt) (Figure 5a,b). Notably, AgNPsE_EtOH3%_-D demonstrated higher cytotoxicity than CisPt (*p* = 4.58 × 10^−3^), while AuNPsEaq-D showed a weaker cytotoxic effect (*p* = 1.82 × 10^−3^). Both gold and silver nanoparticles derived from Dandelion extracts also exhibited strong cytotoxicity against the breast cancer cell line (MDA-MB-231), outperforming doxorubicin (DOX) (χ^2^ = 42.035; *p* = 5.79 × 10^−8^). AgNPsE_EtOH3%_-SW exhibited the highest cytotoxicity (*p* = 1.43 × 10^−8^), followed by AgNPsE_EtOH3%_-D (*p* = 7.20 × 10^−5^) and AuNPsE_EtOH3%_-D (*p* = 4.72 × 10^−4^). These nanoparticles significantly surpassed DOX in tumor cell inhibition, while maintaining normal cell viability.

After 48 h, AgNPsE_ETOH_-SW influenced the breast tumor cell line without impacting normal cells (Figure 5a,b). The E_ETOH30%-_SW extract demonstrated higher cytotoxicity than DOX (*p* = 4.58 × 10^−3^), confirming its potential as a therapeutic agent.

Other studies have also shown a strong antitumor effect of biosynthesized AgNPs, particularly on breast tumor cell lines [58,59]. Additionally, polyphenols from the *Artemisia annua* extract have shown anticancer activity in highly metastatic breast cancer cells (MDA MB-231) [60].

Regarding the liver tumor line, HepG2, the high concentrations of the analyzed samples had a substantial antitumor effect (χ^2^ = 105.868; *p* = 1.67 × 10^−19^), especially in the case of the 30% alcoholic wormwood extract (*p* = 1.59 × 10^−9^ compared to CisPt). The effect became much more pronounced after 48 h, showing a stronger cytotoxic impact than CisPt (Figure 5c).

Silver nanoparticles from wormwood (aqueous and alcoholic extracts) also exhibited significant antitumor effects on HepG2 liver tumor cells, though slightly weaker than those of the wormwood extract. Notably, AuNPsE_EOTH5%_-SW (*p* = 9.19 × 10^−9^ vs. CisPt) and AgNPsEaq-D (*p* = 3.49 × 10^−9^ vs. CisPt) demonstrated a considerable reduction in tumor cell viability. Artemisinin, the phytotherapeutic compound in wormwood, has been widely recognized for its antimalarial and antitumor effects. Drug delivery systems based on artemisinin have already been developed to efficiently and selectively target specific tumor cell lines [61]. Utilizing synergistic effects, green-synthesized nanoparticles from Sweet Wormwood extracts could be a promising strategy to enhance controlled phytoconstituent delivery, while minimizing toxicity risks.

#### 3.6.2. Statistical Analysis

The cytotoxic effects of the tested compounds on different cell lines were statistically analyzed and summarized in Table S7. The Chi-square (χ^2^) test results indicate significant differences between the tested treatments and standard chemotherapeutics across all analyzed cell lines, with high χ^2^ values and extremely low *p*-values confirming the cytotoxic effects of the green-synthesized nanoparticles. This table presents the comparative performance of the studied formulations against control treatments (Cis-Pt and DOX), highlighting statistically significant differences in cytotoxicity. The results indicate that silver nanoparticles from Dandelion and Sweet Wormwood extracts exhibited strong antitumor activity, particularly against MDA-MB-231 and LoVo cell lines, while maintaining lower toxicity towards normal cells (HUVEC). Notably, ethanolic wormwood extract (30%) demonstrated superior efficacy in targeting HepG2 liver cancer cells, outperforming Cis-Pt. These findings further support the potential of plant-based nanoparticles as promising alternatives in cancer therapy.

#### 3.6.3. Comparative Analysis of IC50 and Selectivity Index at 24 h and 48 h

The comparative analysis of the IC50 values and selectivity indices at 24 h and 48 h reveals significant changes in the cytotoxicity and specificity of the tested compounds (Tables S7 and S8 and Figure 6). A notable decrease in IC50 values over time is observed for most compounds, particularly E_EOTH30%_-SW and AuNPsEaq-D, indicating an increase in their potency on cancer cells. This trend is especially pronounced in the case of MDA-MB cells, where E_ETOH30%-_SW shows a drastic reduction in IC50 from 0.06848 (24 h) to 0.02412 (48 h), while AuNPsE_aq_-D decreases from 0.21951 to 0.02800, suggesting a much stronger effect with prolonged exposure. In contrast, AgNPsE_ETOH3%_- SW exhibits a loss of selectivity for MDA-MB over time, as its IC50 increases from 0.04909 (24 h) to 0.21758 (48 h), resulting in a significant drop in its selectivity index from 2.21 to 0.37. This suggests that its cytotoxic effect on cancer cells diminishes with prolonged exposure. Meanwhile, AgNPsE_ETOH3%_-D maintains a stable IC50 for LoVo and sees an improvement in selectivity for MDA-MB, with the selectivity index increasing from 1.32 (24 h) to 2.56 (48 h), indicating an enhancement in its specificity towards breast cancer cells. Among all tested compounds, AuNPsEaq-D demonstrates the most remarkable shift in selectivity, with its selectivity index for MDA-MB skyrocketing from 0.31 at 24 h to 8.41 at 48 h, making it the most promising candidate for targeted breast cancer therapy. This drastic increase suggests that AuNPsEaq-D preferentially kills cancer cells, while sparing normal HUVEC cells, a crucial characteristic of an effective anticancer agent. Similarly, E_ETOH30%_-SW also improves in selectivity over time, with its selectivity index for MDA-MB increasing from 5.92 (24 h) to 7.51 (48 h), reinforcing its potential for cancer treatment. On the other hand, Cis-Pt continues to show poor selectivity across both time points, with a consistently low selectivity index for LoVo (0.14 at 24 h and 0.12 at 48 h) and no selectivity for MDA-MB, confirming its known broad cytotoxicity against both cancerous and healthy cells. These results suggest that while Cis-Pt remains a standard chemotherapy agent, the newly tested compounds, particularly AuNPsE_aq-_D and E_ETOH30%_-SW, offer more promising alternatives with improved selectivity and reduced side effects on normal cells. Overall, the 48-h data provides strong evidence that AuNPsE_aq_-D and E _ETOH30%_- SW are the most effective and selective treatments for MDA-MB cells, while AgNPsE_ETOH30%_-D also shows improved potential. The best samples for HepG2-targeted therapy based on SI values are E_ETOH30%_-SW and AgNPsE_ETOH5%_-D, which show the highest selectivity. Cis-Pt has lower selectivity than some of these nanoparticles, suggesting potential for replacing or combining nanoparticles with standard chemotherapies. Further in vivo studies and normal cell toxicity assessments are needed to validate clinical relevance. The enhanced selectivity over time suggests that these compounds may work more efficiently with prolonged exposure, supporting their potential for further preclinical and clinical studies. Future research should focus on mechanistic studies to understand the pathways involved in their cytotoxic effects and confirm their selectivity using additional cancer models.

#### 3.6.4. The Potential Antitumoral Mechanism

- Effects of Nanoparticles on MDA-MB-231 Breast Cancer Cells

Comparing the AuNPs from *Taraxacum officinale* (dandelion) with those from *Artemisia annua* (sweet wormwood), we can observe that dandelion-derived nanoparticles exhibit a stronger anticancer effect, despite their lower antioxidant activity. This difference can be explained by several factors related to morphology, stability, and specific cellular mechanisms. AuNPs from dandelion have a triangular morphology, which provides a higher surface-to-volume ratio compared to spherical nanoparticles. This results in 1. stronger interaction with tumor cell membranes; 2. increased penetration of cancer cells through endocytosis or more intense contact with biomolecules; 3. influencing intracellular processes [62]. AgNPs from *Artemisia annua* are more stable and have a spherical shape, making them less reactive in their interaction with tumor cells.

AuNPs from dandelion (AuNPsEaq-D and AuNPsE_ETOH_-D), despite not exhibiting strong antioxidant properties, can induce specific oxidative stress in cancer cells. They can disrupt the redox balance of tumor cells, which are already characterized by high oxidative stress levels. Gold nanoparticles are known to interact with mitochondria and deregulate the energetic metabolism of cancer cells [62,63]. Silver nanoparticles from *Artemisia annua* (AgNPsEaq-SW, AgNPsE_ETOH_-SW) exhibit higher antioxidant activity (e.g., AgNPsEaq-SW = 83.10%), suggesting that they may reduce oxidative stress rather than amplify it, which could limit their anticancer potential. Another crucial factor is the instability of dandelion-derived gold nanoparticles, which makes them more reactive in biological environments. This instability results in a faster release of gold ions, allowing for enhanced interactions with proteins and nucleic acids inside cancer cells. In contrast, silver nanoparticles from *Artemisia annua* are more stable and, therefore, may have a slower or less aggressive anticancer action. Finally, the bioactive compounds present in *Taraxacum officinale*, such as flavonoids and terpenoids, work synergistically with gold nanoparticles to amplify oxidative stress and induce apoptosis [64]. While *Artemisia annua* contains artemisinin, a compound known for its reactive oxygen species (ROS)-generating effects, its mechanism relies on iron metabolism, which may not be as effective in combination with silver nanoparticles [65]. The AuNPsEaq-D and AuNPsE_ETOH_-D exhibit higher selectivity for cancer cells compared to DOX, meaning they effectively target tumor cells without harming normal cells. This selective action can be explained by several factors, as follows: cancer cells are more susceptible to ROS stress than normal cells, making AuNPs more effective in targeting tumors [66,67]; nanoparticles enter cancer cells more efficiently than normal cells, reducing off-target effects; normal cells have stronger antioxidant defenses, protecting them from AuNP-induced stress; and unlike DOX, AuNPs do not directly bind to DNA or interfere with normal cell division, reducing systemic toxicity [68].

- Effects of Nanoparticles on Colon Cancer

Silver nanoparticles derived from Taraxacum officinale (AgNPsE_ETOH_-D) exhibit superior anticancer effects against LoVo colon cancer cells, demonstrating greater efficacy than CisPt. This enhanced effectiveness can be attributed to their unique physicochemical properties, which allow for increased cellular uptake, oxidative stress induction, and apoptosis activation, while minimizing toxicity to healthy cells. In contrast, silver nanoparticles from *Artemisia annua* (AgNPsEaq-SW) show comparable anticancer effects to Cis-Pt, but with a significantly higher safety profile, reducing the risk of adverse side effects commonly associated with chemotherapy. These findings suggest that silver nanoparticles from Dandelion and Sweet Wormwood may serve as promising alternatives or complementary agents to conventional colon cancer treatments.

- Effects of Nanoparticles on Liver Cancer (HepG2)

The E_ETOH30%_-SW demonstrates high anticancer efficacy, showing significant cytotoxic effects after 48 h, ultimately surpassing CisPt in its ability to reduce cancer cell viability. This effect can be attributed to the high content of bioactive compounds, particularly artemisinin, which is known to generate reactive oxygen species (ROS) selectively in cancer cells, disrupting their redox balance and leading to apoptosis. Additionally, the presence of flavonoids and terpenoids enhances oxidative stress, further impairing tumor cell survival.

Similarly, the AgNPsE_ETOH3%_-SW induce a substantial decrease in cancer cell survival while exhibiting no toxic effects on normal cells, a selectivity likely driven by the differential redox environment of cancer versus normal cells. Cancer cells, which already experience higher baseline oxidative stress, are more susceptible to ROS-mediated damage induced by silver nanoparticles, whereas normal cells possess stronger antioxidant defense mechanisms that protect them from oxidative stress-related damage. Moreover, the nanoparticles interact with mitochondrial membranes, further destabilizing cancer cell metabolism and triggering apoptosis, making them a promising and selective alternative to conventional chemotherapy, with reduced systemic toxicity compared to CisPt.

**Figure 7:**
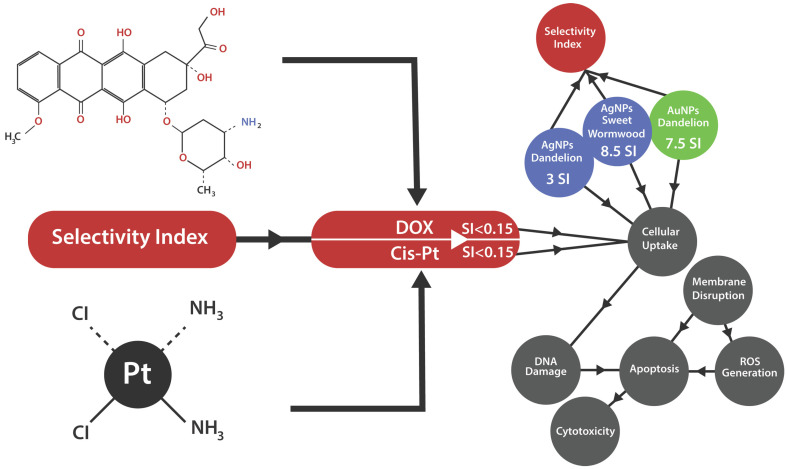
Selectivity Index and mechanisms of action of green-synthesized nanoparticles compared to conventional chemotherapeutic agents.

The following schematic representation in Figure 7 highlights the key mechanisms of nanoparticle action on cancer cell lines. It illustrates processes, such as enhanced cellular uptake, reactive oxygen species (ROS) generation, DNA damage, mitochondrial dysfunction, and apoptosis induction. These mechanisms collectively demonstrate the selective cytotoxicity of the green-synthesized nanoparticles and underscore their potential as targeted cancer therapies with reduced toxicity compared to conventional chemotherapeutics like DOX and CisPt.

### 3.7. Limitations of the Study

Despite the promising findings, this study has several limitations that must be acknowledged. First, the long-term environmental and biological effects of green nanoparticles remain largely unexplored, requiring extended studies to assess their bioaccumulation potential and degradation pathways. Additionally, while in vitro experiments provide valuable insights into cytotoxicity and antimicrobial efficacy [69], they do not fully replicate the complex interactions occurring within living organisms, necessitating in vivo studies for a more comprehensive evaluation. Furthermore, variations in plant-derived bioactive compounds used for nanoparticle synthesis may lead to inconsistencies in physicochemical properties, potentially affecting reproducibility and large-scale production. Another limitation is the lack of detailed mechanistic studies on nanoparticle interactions at the molecular level, which are essential for understanding their potential risks and therapeutic applications. Lastly, while this study emphasizes the eco-friendly nature of green nanoparticles, a full life-cycle assessment is required to determine their overall sustainability, including energy consumption, waste generation, and long-term ecological footprint.

## 4. Materials and Methods

### 4.1. Plant Material and Preparation of the Plant Extract

The aerial parts of Dandelion (*Taraxacum officinale* herba) and Sweet Wormwood (*Artemisia annua* herba) were collected from Ilfov County, Romania, during June–July 2022. Voucher specimens were deposited at the Herbarium of the Bucharest Botanical Garden, University of Bucharest, Faculty of Biology, under reference numbers 410416 and 408939, respectively. The plant materials were washed, shade-dried at room temperature for seven days, and then ground using a mechanical grinder, as shown in Figures S1 and S2 from the Supplementary Materials (SM).

Ethanolic extracts were prepared by mixing 1 g of each dried, sieved plant powder (using sieve no. VI) with 100 mL of ethanol (30% or 50%). The mixtures were heated under reflux on a water bath for 30 min and subsequently filtered through Whatman® qualitative filter paper, Grade 1 (Merck KGaA, Darmstadt, Germany). The resulting extracts were stored at 4 °C until further analysis.

### 4.2. Green Synthesis and Characterization Methods

For the synthesis of silver nanoparticles using Dandelion extracts, 5 mL of ethanolic Dandelion extract (E_ETOH_-D), prepared using ethanol at concentrations of 30% or 50% (*v*/*v*), was added to 50 mL of a 1 mM AgNO_3_ solution (Sigma Aldrich, Darmstadt, Germany). The mixture was incubated at 60 °C under continuous shaking for 30 min. A visible color change to dark brown indicated the successful formation of silver nanoparticles. The suspensions were then stored at 4–8 °C, away from light, until characterization. Similarly, silver nanoparticles were synthesized using the aqueous Dandelion extract (Eaq-D) following the same procedure.

To synthesize gold nanoparticles, 10 mL of Sweet Wormwood extract (extracted in ethanol at 30% or 50% *v/v* concentration, denoted as E_ETOH_-SW) was mixed with 100 mL of 0.5 mM HAuCl_4_·3H_2_O solution (Sigma Aldrich, Darmstadt, Germany). The resulting mixture was incubated at 60 °C for 30 min under continuous shaking. The development of a violet color indicated the formation of gold nanoparticles. The synthesized nanoparticles were then stored at a controlled temperature of 4–8 °C, protected from light, until further characterization. Similarly, gold nanoparticles were synthesized using only the aqueous Sweet Wormwood extract, referred to as AuNPsEaq-SW.

#### 4.2.1. UV-VIS Absorption and Fluorescence

UV-VIS properties of the green synthesis were recorded using both the plant and their corresponding extract using a U-0080D UV–Vis spectrometer (Hitachi, Tokyo, Japan, Japan), while fluorescence emission spectra were recorded with a spectrometer equipped with a 450 W Xenon lamp as an excitation source (model FLS920, Edinburgh Instruments Ltd., Livingston, UK).

UV-VIS spectrophotometric analysis was employed to monitor the formation of silver and gold nanoparticles synthesized via green reduction using *Taraxacum officinale* (Dandelion) and *Artemisia annua* (Sweet Wormwood) extracts. Absorption spectra were recorded in the 200–700 nm range using a UV-Vis spectrophotometer.

Nanoparticles were synthesized by mixing aqueous or ethanolic plant extracts with metal salt solutions. For instance, silver nanoparticles were obtained by adding aqueous Dandelion extract to a 1 mM AgNO_3_ solution in a 1:10 ratio. Similar procedures were followed for the synthesis of gold nanoparticles by a reduction in HAuCl_4_·3H_2_O with both aqueous and ethanolic extracts of Dandelion and Sweet Wormwood.

A theoretical size estimation of gold/silver nanoparticles using the Haiss equation [38] has been moved to the Supplementary Materials.

Fluorescence spectra were recorded using a fluorescence spectrophotometer. Emission was measured in the range of 300–700 nm to capture signals corresponding to polyphenolic compounds (300–380 nm) and chlorophyll derivatives (600–700 nm).

#### 4.2.2. FTIR-ATR

A Bruker Optics Tensor 27 spectrometer equipped with an ATR Platinum accessory with a single reflection diamond crystal was employed for FTIR measurements. All samples were traced after 64 scans, with a resolution of 4 cm^−1^, on a spectral domain of 4000–400 cm^−1^. Each sample was prepared using a drying process at room temperature.

ATR-FTIR (attenuated total reflectance—Fourier transform infrared) spectroscopy was used to identify functional groups in the plant extracts involved in the reduction and stabilization of silver and gold nanoparticles. FTIR spectra were collected for both the alcoholic extracts of Dandelion and Sweet Wormwood, as well as for their respective synthesized nanoparticles.

#### 4.2.3. Scanning Electron Microscopy (SEM)

Nanoparticles were investigated morphologically using Nova NanoSEM 630, a field emission scanning microscope (FE-SEM) (FEI Company, Hillsboro, OR, USA), with an operating voltage of 5 kV and a magnitude of 40 to 300 kx.

#### 4.2.4. High Resolution Transmission Electron Microscopy (TEM)

Sample preparation for transmission electron microscopy was performed using the following sequence. First, Formvar and Carbon-coated 100 mesh copper TEM grids were plasma treated to obtain a hydrophilic grid surface, by means of a PELCO easiGlow™, Glow Discharge System. Then, 1 mL of each sample was pipetted in 1.5 mL Eppendorf tubes and sonicated for 1 to 2 min at room temperature, at 59 kHz, in an ultrasonic bath (FALC LBS 2–4.5 L). Afterword, 4 µL of each of the sonicated contents were pipetted on the treated TEM grids, slightly blotted with filter paper, and left to dry for 10 min. Micrographs were recorded on these grids using the 4 k × 4 k Ceta camera of a Talos F200c transmission electron microscope (Thermo Fisher Scientific, Waltham, MA, USA), at 200 kV, at 92,000×, 240,000×, 390,000×, and 650,000× nominal magnifications.

#### 4.2.5. Hydrodynamic and Electrophoretic Light Scattering Measurements

The hydrodynamic diameter and surface charge of the colloidal dispersions were characterized using a Delsa™ Nano C instrument Delsa™ Nano C (Beckman Coulter, Brea, CA, SUA) by DLS and electrophoretic light scattering (ELS). Particles were illuminated by a dual 30 mW laser diode, producing time-dependent fluctuations in the intensity of the laser light. The scattered light was collected at 165° for size measurements and at 15° for zeta potential measurements (for diluted concentration samples) and then recorded by a highly sensitive detector.

Both DLS and ELS measurements were carried out at room temperature, and each analysis was performed in triplicate. The resulting data was processed using the Delsa™ Nano 3.73 software package.

The polydispersity index (PDI) was also determined for each colloidal sample, as a measure of size distribution uniformity. The PDI represents the ratio of particles of varying sizes relative to the total population, with values closer to zero (<0.1) indicating a monodisperse system and higher values suggesting increased heterogeneity in nanoparticle size distribution.

#### 4.2.6. Total Polyphenol Content and Total Flavonoids Content

The total polyphenol content was determined using the Singleton colorimetric method based on the Folin–Ciocalteu reagent, with gallic acid as the standard. The total flavonoid content was measured by the aluminum chloride colorimetric method according to the Romanian Pharmacopoeia [70,71]. Results were expressed as mg gallic acid equivalent (GAE)/g dry weight for polyphenols and mg rutin equivalent (RE)/g dry weight for flavonoids. All assays were performed in triplicate. These methods quantify phenolic compounds involved in the green synthesis of nanoparticles. Further details on the experimental procedures and reagent specifications are provided in the Supplementary Materials.

### 4.3. Description of Samples and Their Abbreviations

All abbreviations used in this study to define the treatments applied to the three tumor cell lines (MDA-MB-231—human breast adenocarcinoma, HepG2—human liver cancer, LoVo—human colon adenocarcinoma) and to the normal human endothelial cell line (HUVEC) are listed and explained below in Table 4.

### 4.4. Biological Tests

#### 4.4.1. Antioxidant Activity

The antioxidant activity of the two indigenous plants was analyzed using the 2,2-diphenyl-1-picrylhydrazylradical scavenging assay. The method is based on the change in color of the DPPH solution from violet to yellow when it is reduced by a proton donor (an antioxidant). For this analysis, 100 mg of DPPH (Sigma Aldrich, Darmstadt, Germany) was diluted with methanol in a 200 mL volumetric flask. In total, 1 mL of the sample was mixed with 5 mL of the diluted DPPH and methanol up to 25 mL. After incubating for 30 min in the dark at room temperature, the absorbance was measured spectrophotometrically at a wavelength of 517 nm. The scavenging ability of the two extracts was calculated using the DPPH scavenging activity % Formula (1):

(1) $$DPPH\%=\frac{{Abs}_{control}-{Abs}_{sample}}{{Abs}_{control}}\times 100$$

where Abs_control_ is the absorbance of diluted DPPH; Abs_sample_ is the absorbance of the sample extracts with DPPH [72,73]. Each assay was performed in triplicate.

#### 4.4.2. Antibacterial and Antifungal Activity

The antibacterial and antifungal activities of the plant extracts and green-synthesized nanoparticles were evaluated using the agar diffusion method, following standardized protocols adapted to comply with current regulatory guidelines [41]. All experimental procedures were conducted under Good Microbiological Laboratory Practice conditions [74,75]. To interpret the results obtained from the inhibition zones, a conventional scale was used as follows: resistant (0 mm—no effect), weak effect (zone < 10 mm), medium effect (10–20 mm), strong effect (over 20 mm), and very strong effect (over 30 mm).

- Microorganisms and Culture Conditions

The tested microorganisms were sourced from the international ATCC collection, the sub-collection of environmental (wild) strains, or clinical isolates available at the Pharmaceutical Microbiology Laboratory. The bacterial strains included *Staphylococcus aureus* (Gram-positive coccus), *Escherichia coli* (Gram-negative bacillus, *Enterobacteriaceae* family), *Pseudomonas aeruginosa* (Gram-negative bacillus, non-Enterobacteriaceae family), and Bacillus subtilis (Gram-positive bacillus, *Bacillaceae* family). For antifungal testing, yeast strains (*Saccharomyces cerevisiae*, *Candida albicans)* and filamentous fungi (*Penicillium* sp., *Aspergillus* sp.) were selected.

Bacterial cultures were grown on Mueller–Hinton agar (MHA), while fungal strains were cultured on Sabouraud Dextrose agar (SDA). All media were inoculated using sterile swabs with standardized microbial suspensions. For each Petri dish (Φ = 10 cm), six sterile cellulose biodiscs (10 µL of test substance each), and six sterile stainless-steel microcylinders (100 µL each) were applied using a calibrated template to ensure uniform sample placement.

- Incubation and Assessment of Antimicrobial Activity

Bacterial plates were incubated aerobically at 37 °C, and inhibition zones were measured after 24, 48, and 72 h. Fungal plates were incubated aerobically at 23–26 °C for periods ranging from 24 h to 7 days, depending on species. The antimicrobial effect was assessed by measuring in millimeters the diameter of the microbial inhibition zones (IZ) surrounding each applied sample. Measurements were taken in two perpendicular directions (maximum and minimum) and expressed as an arithmetic mean with a precision of 0.1 mm. The minimum inhibitory concentration (MIC) was calculated and reported in µg/µL.

- Sterility and Microbial Load Evaluation

The microbial sterility and inherent microbial load of the extracts were also evaluated by determining bacterial and fungal colony-forming units (CFUs), along with specific physicochemical and antimicrobial tests. These analyses helped confirm the sterility and intrinsic antimicrobial activities of the tested formulations. All experiments were performed using the infrastructure, equipment, laboratory glassware, consumables, reagents, and protective materials provided by the Pharmaceutical Microbiology Laboratory.

The MIC was calculated in μg/μL (equivalent to mg/mL or g/L) for each substance on each microbial strain by evaluating the volume (V) into which the substance diffused, using Formula (2) where R is the radius of the IZ (half the diameter), and h is the thickness of the culture medium.

(2) $$V=\pi \cdot R^{2}\cdot h$$

#### 4.4.3. Antitumoral Tests

- Sample Preparation

For each of the studied plant extracts—Dandelion (D) and Sweet Wormwood (SW)—both aqueous and ethanolic extracts were subjected to serial dilutions in deionized water using the following ratios: 1:1, 1:2, 1:4, 1:8, 1:16, 1:32, 1:64, and 1:128. The same dilution scheme was applied to samples containing biosynthesized AgNPs and AuNPs. Two reference drugs, DOX and CisPt, were included as positive controls, prepared under identical dilution conditions starting at 10 mg/mL. All cytotoxicity results obtained for each extract or nanoparticle–extract complex were compared against the effects of these two clinically approved chemotherapeutic agents.

- Cell Lines and Culture Conditions

The antitumoral potential of the test substances was evaluated using four human cancer cell lines and one normal endothelial cell line, as follows: MDA-MB-231 (human breast adenocarcinoma; ECACC, Cat. No. 92020424), LoVo (human colorectal adenocarcinoma; ATCC, Cat. No. CCL-229™), HepG2 (human hepatocellular carcinoma; ATCC, Cat. No. HB-8065™), and HUVEC (human umbilical vein endothelial cells; ATCC, Cat. No. CRL-1730™). Cells were cultured in DMEM/F12 medium, supplemented with 10% fetal bovine serum (FBS), 2 mM L-glutamine, 100 U/mL penicillin, and 100 μg/mL streptomycin (Sigma Aldrich, St. Louis, MO, USA). Cultures were maintained at 37 °C in a humidified 5% CO_2_ atmosphere. Cells were seeded into culture flasks and allowed to adhere until ~60% confluence was reached. After 24 h, cells were treated with the prepared dilutions for predetermined time periods. Untreated cells were designated as negative controls. For subculturing, cells were detached using a PBS/1 mM EDTA solution, washed twice in PBS, and subsequently used for cytotoxicity assays.

- MTS Cytotoxicity Assay

The cytotoxicity of the samples was evaluated using the CellTiter 96^®^ Aqueous One Solution Cell Proliferation Assay (Promega Corporation, Madison, WI, USA), based on the MTS [3-(4,5-dimethylthiazol-2-yl)-5-(3-carboxymethoxyphenyl)-2-(4-sulfophenyl)-2H-tetrazolium] colorimetric method. This assay quantifies the metabolic activity of viable cells via their ability to convert MTS to soluble formazan. Approximately 1 × 10^4^ cells/well were seeded in 100 µL medium in flat-bottom 96-well plates (Falcon, Teterboro, NJ, USA), incubated for 24 h, and then exposed to various concentrations of test or control substances for 24 h or 48 h. After treatment, 20 µL of MTS reagent was added per well, and the plates were incubated for 4 h at 37 °C, with gentle shaking every 20 min. The absorbance was recorded at λ = 492 nm using a Dynex ELISA reader (DYNEX Technologies–MRS, Chantilly, VA, USA).

- Data Analysis

Cell viability was calculated relative to untreated control cells (considered 100% viable), using the following equation:

(3) $$Cell\;lysis(\%)=100-\frac{T-B}{U-B}\times 100$$

where T = absorbance of treated cells, U = absorbance of untreated cells, and B = absorbance of blank (culture medium only), measured at 492 nm. All experiments were performed in triplicate, and results are reported as mean ± standard deviation (SD) from three independent experiments.

#### 4.4.4. Statistical Analysis

Statistical analysis was carried out using Python 3.12 to evaluate the statistical differences of the prepared compounds (E_EOTH30%_-SW, AgNPsE_EOTH3%_-SW, AgNPsE_EOTH3%_-D, AuNPsE_aq_-D and AuNPsE_EOTH3%_-D) in comparison with the control ones (Cis-Pt, DOX) on a per cell type (HUVEC, LoVo, MDA-MB, HepG2) basis. The assumption of continuity of variables and the absence of outlier conditions for statistical analysis have been observed; however, due to the dependence of samples (dilutions, measurements at 24 h and 48 h), a repeated measure statistical method needed to be employed. Moreover, with Kolmogorov–Smirnov test for data normality being invalidated (*p* < 0.05), a non-parametric statistical test has been decided upon (Friedman test) followed by a post hoc test (Siegel-Castellan) to assess the source of the statistical difference. Analysis has been implemented using the scipy.stats and scikit_posthoc libraries. Statistical significance was set at α = 0.05, with results being considered significant when *p* < α.

#### 4.4.5. Determination of Half Maximal Inhibitory Concentration and Selectivity Index

The Half Maximal Inhibitory Concentration (IC50) value was determined using Python 3.12 by fitting a four-parameter logistic regression (4PL) curve to the data through the scipy curve_fit function. IC50 represents the concentration at which 50% of the cells are inhibited compared to untreated controls. These curves depict variations in cell survival, reflecting either increased cytotoxicity or reduced proliferation. The half-maximal inhibitory concentration (IC50), which indicates the concentration required to achieve a 50% reduction in viable cells, is a key parameter for evaluating the pharmacological and biological effects of a treatment. IC50 values are typically reported as mean ± standard error, with error estimation derived from a four-parameter logistic (4PL) regression model. In this study, the regression was performed using Python, specifically utilizing the scipy.optimize.curve_fit function. The Selectivity Index (SI) was calculated to determine how selective each compound was for cancer cells relative to normal cells [76]. The following formula (4) used was:

(4) $$SI=\frac{{IC50}_{HUVEC}}{{IC50}_{Cancer\;cell}}$$

Higher SI values suggest greater selectivity for tumor cells, while lower values indicate a less favorable therapeutic window. If SI > 2–3, it indicates high selectivity for cancer cells (desirable). SI ≈ 1 correlates to no selectivity, affecting both normal and cancer cells. Finally, for SI < 1, the compounds are more toxic to normal cells than to cancer cells (undesirable). Each compound’s SI was calculated for both LoVo and MDA-MB cells using HUVEC IC50 values as a reference.

## 5. Conclusions

Antioxidant and Antimicrobial Potential: AgNPsEaq-SW demonstrated the highest antioxidant and antibacterial activity, making it a strong candidate for wound healing and antimicrobial coatings.

Selective Cytotoxicity and Fluorescence-Based Stability: triangular AuNPsEaq-D and AuNPsE_ETOH_-D showed enhanced cellular uptake, supporting their potential use in anticancer therapies, while fluorescence signals confirmed the role of polyphenols in nanoparticle stabilization.

Bioactivity Correlations: the observed fluorescence intensity and stability trends in AuNPs correlate with their ability to retain bioactive compounds, improving their targeted therapeutic potential.

Dandelion-Derived Nanoparticles: despite lower antioxidant activity, they displayed notable antibacterial effects, highlighting their potential in microbial control applications.

Influence of Ethanol Percentage: The presence of ethanol at 3% and 5% concentrations significantly influenced nanoparticle formation, stability, and biological activity. Ethanol enhanced flavonoid extraction, leading to higher polyphenol content and increased fluorescence stability. However, ethanol also impacted nanoparticle aggregation and colloidal stability, with higher ethanol content (5%) favoring smaller, more stable nanoparticles, whereas lower ethanol (3%) resulted in moderate aggregation but retained bioactive compounds more effectively.

Shape and Size Effects on Bioactivity: The morphology and size distribution of nanoparticles significantly influenced their biological activities. Triangular AuNPs exhibited enhanced cellular uptake and cytotoxicity, suggesting that shape anisotropy may improve interaction with cancer cells. Similarly, smaller and more uniformly distributed AgNPs correlated with stronger antibacterial effects. These findings underline the critical role of physical parameters—such as size and shape—in optimizing nanoparticle design for biomedical applications.

- Future Research Directions

In Vivo Testing for Therapeutic Validation: further research should evaluate the pharmacokinetics, biodistribution, and toxicity of these nanoparticles in animal models.

Optimization for Drug Delivery: enhancing targeted delivery mechanisms using functionalized nanoparticles could improve their selectivity in cancer therapy.

Exploring Synergistic Effects: Investigating combination therapies with existing antibiotics and chemotherapeutic agents may enhance their efficacy while reducing resistance. Our previous study demonstrated the synergistic effect of green nanoparticles in combination with chemotherapeutic drugs on HepG2 cells, supporting further investigations into other tumor cell lines [77].

Further Ethanol Influence Studies: More research should explore optimal ethanol concentrations for maximizing nanoparticle stability, bioactivity, and therapeutic efficiency, while minimizing aggregation effects.

Life Cycle Impact of Green Nanoparticles: Green nanoparticle synthesis offers a more sustainable approach, minimizing toxic waste and energy consumption, while enhancing environmental and biological safety. Compared to conventional methods, AgNPs have a greener life cycle, reducing pollution, energy use, and potential toxicity. However, while their organic coatings degrade naturally, the metallic cores may persist, highlighting the need for further studies on their long-term fate and environmental interactions. Additionally, scalability remains a challenge, requiring the optimization of biological synthesis for industrial applications without compromising nanoparticle quality.

- Final Remarks

This study highlights the potential of plant-based nanoparticles as multifunctional therapeutic agents, with antimicrobial, antioxidant, and anticancer applications. The comparison with conventional drugs, such as Cis-Pt and DOX, suggests that these nanoparticles could serve as alternative or complementary agents in chemotherapy, with potentially lower systemic toxicity. Future studies should focus on enhancing stability, optimizing formulations for targeted delivery, and validating in vivo efficacy to bring these nanoparticles closer to clinical applications.

## Figures and Tables

**Table 1:** Total polyphenol (gallic acid, GAE) and flavonoid (rutin, RE) contents.

Sample	TPC (mg GAE/g DW)	TFC (mg RE/g DW)
Extracts		
E_ETOH_-D	15.78 ± 0.012	7.95 ± 0.01
Eaq-D	45.03 ± 0.039	7.42 ± 0.02
E_ETOH_-SW	16.82 ± 0.02	9.17± 0.03
Eaq-SW	44.82 ± 1.99	6.16 ± 0.03
NPs Au/Ag *Artemisia herba* (Romanian Sweet Wormwood)		
	TPC (µg GAE/mL)	TFC (µg RE/mL)
Ag NPsEaq-SW	97.90 ± 14.68	20.2 ± 2.42
AgNPsE_ETOH_-SW	81.30 ± 12.20	35.81 ± 4.29
Au NPsEaq-SW	77.54 ± 11.63	35.23 ± 4.22
Au NPsE_ETOH_-SW	55.40 ± 8.31	152.15 ± 18.25
NPs Au/Ag *Taraxacum herba* (Romanian Dandelion)		
Ag NPs Eaq-D	33.15 ± 4.97	-
AgNPs E_ETOH_-D	34.52 ± 5.18	18.96 ± 2.27
AuNPs Eaq-D	40.39 ± 6.05	-
AuNPs E_ETOH_-D	71.46 ± 10.72	-

**Table 2:** Hydrodynamic diameter (nm), polydispersity index (PDI), and zeta potential (mV) of green nanoparticles in colloidal solution.

Sample #	PDI *	NP Hydrodynamic Size (nm)	Zeta Potential (mV)
NPs Au/Ag *Artemisia annua*			
Ag NPs Eaq-D	0.158	337.4	−0.31
AgNPs E_ETOH_-D	0.258	500	−125.82
AuNPs Eaq-D	0.264	202.8	−0.36
AuNPs E_ETOH_D	0.268	245.6	−0.36
NPs Au/Ag *Taraxacum officinale*			
Ag NPsEaq-SW	0.267	71.1	−41.95
AgNPsE_ETOH_-SW	0.158	90.5	−57.14
Au NPsEaq-SW	0.197	436.5	−48.14
Au NPsE_ETOH_-SW	0.653	449.9	−53.29

* PDI: polydispersity index; # Sample where use without any dilution after biosynthesis.

**Table 3:** The green NPs size and morphology determined by TEM.

Sample	TEM	Morphology/Shape
Mean ± SD (nm)		
AgNPsEaq-SW	11.7 ± 4.6	Spherical
Au NPsEaq-SW	20.4 ± 6.6	Spherical, triangular-shaped, and rod-like structures (canes)
AuNPsEaq-D	26.9 ± 14.3	Triangular particles
AgNPs Eaq-D	9.5 ± 4.0	Spherical
AgNPsE_ETOH_-SW	12.2 ± 4.5	Spherical
AuNPsE_ETOH_-SW	12.8 ± 3.4	Spherical, triangular-shaped, and rod-like structures (canes)
AuNPs E_ETOH_-D	9.8 ± 2.7	Triangular particles
AgNPs E_ETOH_D	15.6 ± 5.9	Spherical

**Table 4:** Sample abbreviations for Dandelion and Sweet Wormwood extracts.

Abbreviation	Description
Eaq-D	Aqueous dandelion extract
E_ETOH_-D	Ethanolic dandelion extract
Eaq-SW	Aqueous sweet wormwood extract
E_ETOH_-SW	Ethanolic sweet wormwood extract
D-NPs	Dandelion-derived nanoparticles (D-NPs)
AgNPsEaq-D	Silver nanoparticles from aqueous dandelion extract
AgNPsE_ETOH_-D	Silver nanoparticles from ethanolic dandelion extract
SW-NPs	Sweet Wormwood-derived nanoparticles
AgNPsEaq-SW	Silver nanoparticles from aqueous sweet wormwood extract
AgNPsE_ETOH_-SW	Silver nanoparticles from ethanolic sweet wormwood extract
AuNPsEaq-D	Gold nanoparticles from aqueous dandelion extract
AuNPsE_ETOH_-D	Gold nanoparticles from ethanolic dandelion extract
AuNPsEaq-SW	Gold nanoparticles from aqueous sweet wormwood extract
AuNPsE_ETOH_-SW	Gold nanoparticles from ethanolic sweet wormwood extract
Mikrozid	Mikrozid^®^ (60% alcohol-based)- positive control
CisPt	Cisplatin
DOX	Doxorubicin

Note: If nanoparticles were obtained from an extract containing 50% or 30% ethanol, the ethanol concentration present in the colloidal nanoparticle solution is indicated next to ETOH (e.g., E_ETOH50%_-D for ethanolic dandelion extract containing 50% ethanol).

## Data Availability

Data supporting the findings of this study can be obtained from the corresponding author upon justified request.

## Supplementary Materials

The following supporting information can be downloaded at https://www.mdpi.com/article/10.3390/ijms26147022/s1.
