# Supplementary material for: Harnessing Plant-Based Nanoparticles for Targeted Therapy: A Green Approach to Cancer and Bacterial Infections
Source: Int J Mol Sci. 2025 Jul 21;26(14):7022. doi: 10.3390/ijms26147022 (PMC12296188; doi:10.3390/ijms26147022)
Supplement: Supplementary file 1 [file ijms-26-07022-s001.zip › ijms-3765438-supplementary.pdf]

Supplementary Material

Article

Harnessing Plant-Based Nanoparticles for Targeted Therapy: A Green Approach to Cancer and Bacterial Infections

**Mirela Claudia Rîmbu [1,2], Daniel Cord [1,2,*], Mihaela Savin [3], Alexandru Grigoroiu [3], Mirela Antonela Mihăilă [1,4], Mona Luciana Gălățanu [1], Viorel Ordeanu [1], Mariana Panțuroiu [1], Vasilica Țucureanu [3], Iuliana Mihalache [3], Oana Brîncoveanu [3], Adina Boldeiu [3], Veronica Anăstăsoaie [3], Carmen Elisabeta Manea [1,5],
Roxana-Colette Sandulovici [1], Marinela Chirilă [1], Adina Turcu-Știolică [6], Emilia Amzoiu [7], Victor-Eduard Peteu [8,9], Cristiana Tănase [10,11], Bogdan Firtat [3] and Carmen-Marinela Mihăilescu [1,3,12*]**

[1] Pharmacy Faculty, "Titu Maiorescu" University, 040314 Bucharest, Romania; mirela.rimbu@prof.utm.ro (M.C.R.); mirela.mihaila@prof.utm.ro (M.A.M.); luciana.galatanu@prof.utm.ro (M.L.G.); ordeanu_viorel@yahoo.com (V.O.); mariana.panturoiu@prof.utm.ro (M.P.); carmen.manea@nipne.ro (C.E.M.); roxana.sandulovici@prof.utm.ro (R.-C.S.); marinela.chirila@prof.utm.ro (M.C.)
[2] Medical Doctoral School, Titu Maiorescu University , 040317 Bucharest, Romania
[3] National Institute for Research and Development in Microtechnologies (IMT Bucharest),
072996 Bucharest, Romania; mihaela.savin@imt.ro (M.S.); alexandru.grigoroiu@imt.ro (A.G.);
vasilica.tucureanu@imt.ro (V.Ț.); iuliana.mihalache@imt.ro (I.M.); oana.brincoveanu24@gmail.com (O.B.); adina.boldeiu@imt.ro (A.B.); veronica.anastasoaie@gmail.com (V.A.); bogdan.firtat@imt.ro (B.F.)
[4] Stefan S. Nicolau Institute of Virology, 030304 Bucharest, Romania
[5] Horia Hulubei National Institute for R&D in Physics and Nuclear Engineering, 30 Reactorului Street,
077125 Magurele, Romania
[6] Pharmacoeconomics Department, University of Medicine and Pharmacy of Craiova, 200349 Craiova, Romania; adina.turcu@umfcv.ro
[7] Department of Physical Chemistry, University of Medicine and Pharmacy of Craiova, 200349 Craiova, Romania; emanro2002@yahoo.com
[8] Ultrastructural Pathology and Bioimaging Laboratory, Institute of Pathology Victor Babeș, Splaiul Independentei 99-101, 050096 Bucharest, Romania; peteuvictoreduard@gmail.com
[9] Faculty of Chemical Engineering and Biotechnologies, Politehnica University of Bucharest,
011061 Bucharest, Romania
[10] Victor Babes National Institute of Pathology, 050096 Bucharest, Romania; cristianatp@yahoo.com
[11] Department of Cell Biology and Clinical Biochemistry, Titu Maiorescu University, 031593 Bucharest, Romania
[12] Doctoral School, University of Medicine and Pharmacy of Craiova, 200349 Craiova, Romania
* Correspondence: daniel.cord@prof.utm.ro (D.C.); carmen.mihailescu@imt.ro (C.-M.M.)

Figure S1. The aerial parts of *Taraxacum officinale* Wigg., Dandelion (D)

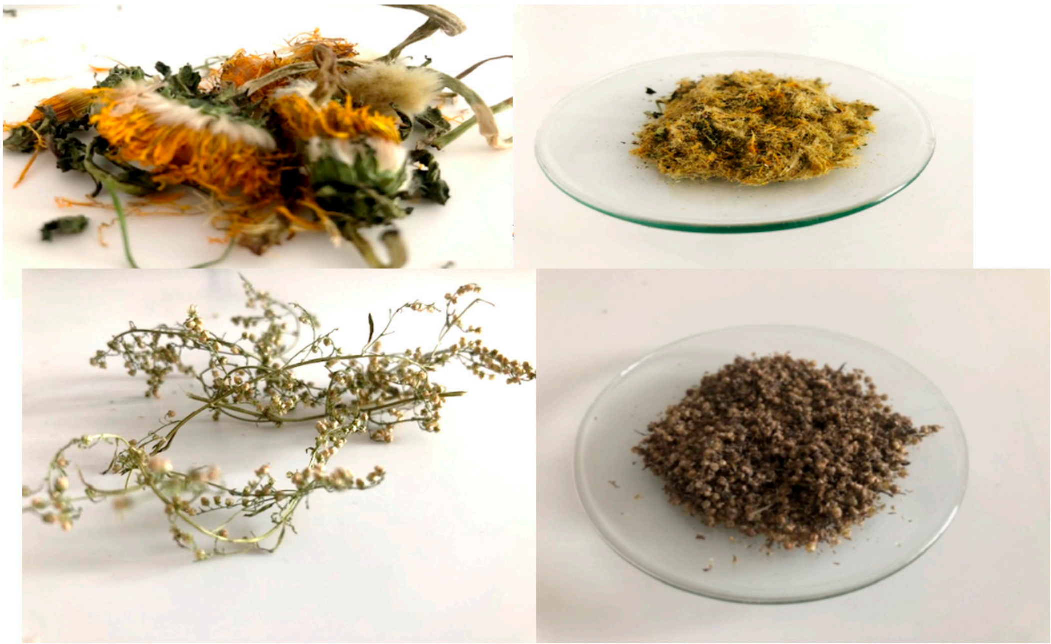

1. Theoretical estimation of gold/silver nanoparticles

Although originally developed for gold nanoparticles, the Haiss equation was applied here to silver colloids as an empirical approximation of particle size based on UV–Vis spectral features.
For gold nanoparticles, theoretical size estimation was performed using the Haiss equation:

$$\boldsymbol{d_{nm}} = \frac{\ln \frac{A_{SPR}}{A_{450}}}{0.0216}$$

Values of maximum absorbance (A_{spr}) and absorbance at 450 nm (A_{450}) were used to calculate nanoparticle diameters.
It is important to note that although the Haiss model offers a simple method for nanoparticle size estimation based on UV–Vis data, it assumes spherical particles and does not account for aggregation or polydispersity—common features of biogenic nanoparticles. Thus, its use in complex colloidal systems such as plant-mediated syntheses must be interpreted with caution. In this study, UV–Vis fitting techniques provided more reliable estimations for AgNPs than for AuNPs, in agreement with prior reports that highlighted a better correlation between UV–Vis spectra and microscopy-based size measurements in silver systems.

2. Determination of Total Polyphenol Content (TPC)

For this determination, 1 mL of plant extract was mixed with 5 mL of Folin-Ciocalteu reagent (previously diluted 10 times with distilled water) and 4 mL of 7.5% sodium carbonate solution (Merck Group, Darmstadt, Germany), with the mixture then stirred and left to stand for 60 minutes in the dark at room temperature. The absorbance of the blue mixture solution was read on a VWR UV-6300 PC Spectrophotometer (VWR International, Wien, Austria) at a wavelength (λ) of 765 nm. A calibration curve was constructed using gallic acid solutions in methanol (Sigma Aldrich, Germany) ranging from 10 to 50 μg/mL, resulting in the following regression equation :

$$C_{TPC} = 25.1359 * A \quad (1) \text{ with } R^2 = 0.9994$$

The results were expressed as mg gallic acid equivalent/g dry matter. Each assay was performed in triplicate. This method quantifies the total phenolic content, compounds that are known to participate in the green synthesis of nanoparticles through reduction and stabilization mechanisms.

3. Determination of the total flavonoid content

The total flavonoid content (TFC) of the two extracts was determined by the aluminum chloride colorimetric method described in the tenth Edition of the Romanian Pharmacopoeia , such that plant extracts (10 mL) were diluted with methanol in a 25 mL volumetric flask and then filtered. Subsequently, 5 mL of these diluted extracts were mixed with 5 mL sodium acetate (100 g/L) (Sigma Aldrich, Darmstadt, Germany), 3 mL aluminum chloride (25 g/L) (Merck Group, Darmstadt, Germany), and methanol to volume 25 mL. The mixtures were kept in the dark for 15 min at room temperature and their absorbances were measured on a VWR UV-6300 PC Spectophotometer (VWR International, Wien, Austria), at a wavelength (λ) of 430 nm, in comparison with a blank prepared in the same way but without the reagents. The concentration of flavonoids was expressed as mg rutin (RE) equivalent/g dry plant material for extracts and in μg/mL for colloidal solution with nanoparticles. The results were expressed as mg rutin equivalent (RE)/g DW for dry plant extracts, and in μg/mL for colloidal nanoparticle solutions. All the regents were of analytical grade, and the solvents were of HPLC quality.

Table S1: The possible assignments of the spectral bands for the samples wormwood extract, dandelion extract and silver and gold nanoparticles obtained using the two types of extracts

Possible bond assignments	From	Wavenumber (cm^{-1})					
		SW dried extract	D extract	AgNPsSW	AuNPsSW	AgNPs D	AuNPs D
**O-H/N-H, ν**	Alcohols or phenols hydroxyl groups over lap amine compound	3268	3274	3243	3275	3290	3279
**C-H, ν**	Aliphatic groups	2916	2930	2927	2929	2933	2932
**C-H, ν**	Aliphatic groups	-	2885	-	-	2886	2886

Possible bond assignments	From	Wavenumber (cm^{-1})					
		SW dried extract	D extract	AgNPsSW	AuNPsSW	AgNPs D	AuNPs D
**C-H, ν**	Aliphatic groups	2850	-	2856	2854	-	-
**C=O, ν**	CO conjugated to the aromatic ring	1766	-		1766	-	-
**C=O+C=C, ν**	CO conjugated to the aromatic ring	1588	1591	1586	1586	1608	1593
**C-O/C-N, ν**	Phenols compounds or aromatic amines	1390	1400	-	-	1409	1396
**C-H, δ**	CH legat de o grupare alcoolica sau aminica	-	1342	1346	1346	1344	-
**C-O++C-H**	Aromatic ethers	1262	1256	-	-	-	1259
**C-O**	Alkyl substituted ether	1108	-	-	-	-	-
**C-O+C-H**	Cyclic ethers	1070	-	1067	1067	-	-
**C-O, δ**	Alcohols or aliphatic ethers	1032	1024	1056	1050	1018	1028
**C-H, δ**	Alcohols or aliphatic ethers	-	926		-	-	922
**C-H, δ**	Cyclohexane ring	897	-	896	896	-	-
**O-O**	Peroxides	872	866	862	868	-	865
**C-H, δ**	Aromatic ring		817	828	826	-	817
**C-H, δ**	Terpenoids	803	-	-	-	-	-
**C-H, δ**	Aromatic ring	-	777	-	-	780	776
**C-H, δ**	Aliphatic groups	669	-	-	-	-	-
**C-H, δ**	Aliphatic groups	618	-	614	612	611	-
**C=C, ν**	skeletal vibration	-	590	-	-	-	-

Possible bond assignments	From	Wavenumber (cm^{-1})					
		SW dried extract	D extract	AgNPsSW	AuNPsSW	AgNPs D	AuNPs D
**O-H, δ**	Alcohols and phenols hydroxyl groups	-	518	-	-	-	519
**C=C, ν**	skeletal vibration	-	421	-	-	437	421
**C=C, ν**	skeletal vibration	-	407	-	-	-	-

Note: ν - stretching and δ - bending vibrations

4. Florescence proprietes

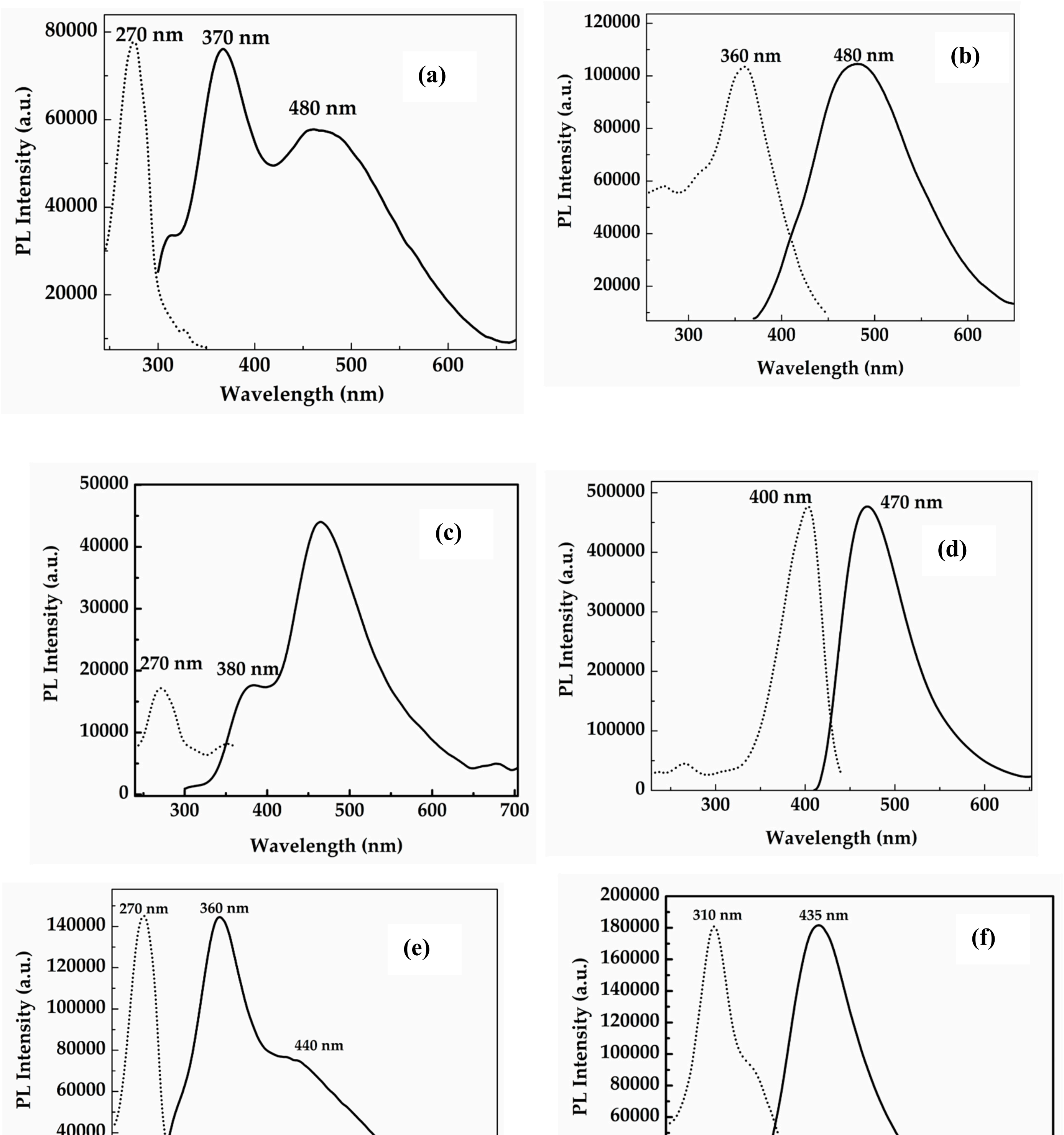

Figure S2. Photoluminescence excitation (PLE) and emission (PL) spectra recorded for **AgNPs E$_{ETOH}$D at** (a) excitation 270nm; (b) 310nm; Photoluminescence excitation (PLE) and emission (PL) spectra recorded for **AgNPsEaqD** at (c) excitation 270 nm and (d) 360 nm excitation; Photoluminescence excitation (PLE) and emission (PL) spectra recorded for **AuNPsE$_{ETOH}$D** at : 270nm (e) excitation and (f) 400nm excitation

Figure S3. Hydrodinamic diameter, polydispersity index and Zeta Potential for: Figure S3.1 Ag/AuNPs SW and Figure S3.2 Ag/Au NPs *D:*

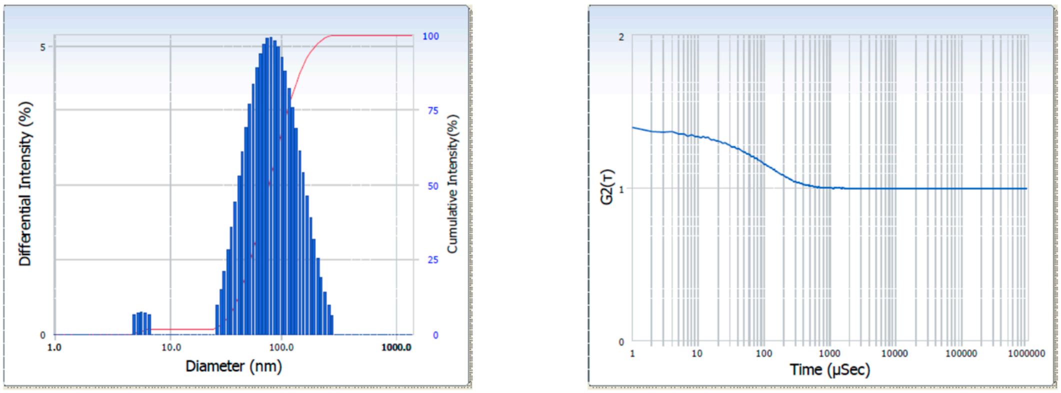

Distribution Results (Contin)

Peak	Diameter (nm)	Std. Dev.

Cumulants Results

Diameter (d) : 71.1 (nm)

Polydispersity Index (P.I.) : 0.267

Figure S3.1(a1) Hydrodinamic diameter, polydispersity index for AgNPsEaq-SW

Version 3.73 / 2.30

Measurement Condition

Sampling Time	: 400	(µs)	Correlation Method	: TD	
Correlation Channel	: 512	(ch)	Accumulation times	: 10	(times)
Scattering Angle	: 15.0	(°)	Temperature	: 25.0	(°C)
Intensity	: 66084	(cps)	Attenuator 1	: 1.12	(%)
Cell Center	: X: 6.710	(mm)	Attenuator 2	: 4.420	(%)
	Z: 6.825	(mm)	Pinhole	: 50	(µm)
Cell Constant	: 105.530	(1/cm)			
Cell Type	: Flow Cell				
Apply Voltage Type	: NEGATIVE				

Electric Field

Avg. Electric Field	: -10.82	(V/cm)	Avg. Current	: -0.10	(mA)

Diluent Properties

Diluent Name	: WATER		Dielectric Constant	: 78.2	
Refractive Index	: 1.3328		Viscosity	: 0.8878	(cP)

Analysis Results

Zeta Potential	: -41.95	(mV)	Zeta Potential of Cell (Upper)	: -29.64	(mV)

FigureS3.1(a2) Zeta Potential for AgNPsEaq-SW

Intensity Distribution

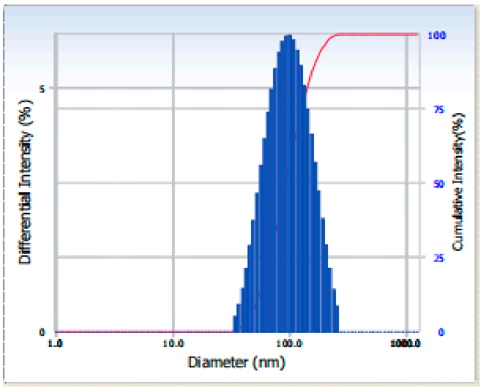

ACF

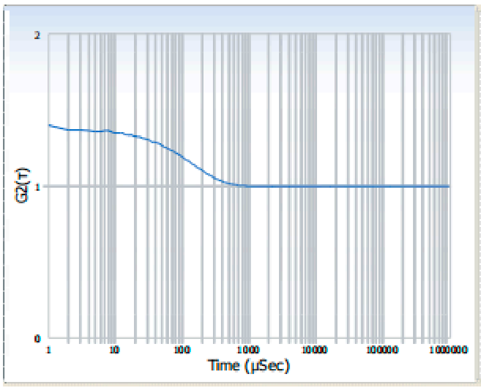

Distribution Results (Contin)

Peak	Diameter (nm)	Std. Dev.

Cumulants Results

Diameter	(d)	: 90.5	(nm
Polydispersity Index (P.I.)		: 0.158	

Figure S3.1(b1) Hydrodinamic diameter, polydispersity index for AgNPsEETOHSW

Version 3.73 / 2.30

Measurement Condition

Sampling Time	: 400	(µs)	Correlation Method	: TD	
Correlation Channel	: 512	(ch)	Accumulation times	: 10	(times)
Scattering Angle	: 15.0	(°)	Temperature	: 25.1	(°C)
Intensity	: 78454	(cps)	Attenuator 1	: 4.2	(%)
Cell Center	: X: 6.710	(mm)	Attenuator 2	: 5.510	(%)
	Z: 6.945	(mm)	Pinhole	: 50	(µm)
Cell Constant	: 187.620	(1/cm)			
Cell Type	: Flow Cell				
Apply Voltage Type	: NEGATIVE				

Electric Field

Avg. Electric Field	: -6.06	(V/cm)	Avg. Current	: -0.32	(mA)

Diluent Properties

Diluent Name	: WATER		Dielectric Constant	: 78.2	
Refractive Index	: 1.3328		Viscosity	: 0.8858	(cP)

Analysis Results

Zeta Potential	: -57.14	(mV)	Zeta Potential of Cell (Upper)	: -64.73	(mV)

Figure S3.1(b2) Zeta Potential for AgNPsEtOHSW

Version 3.73 / 2.30

Intensity Distribution

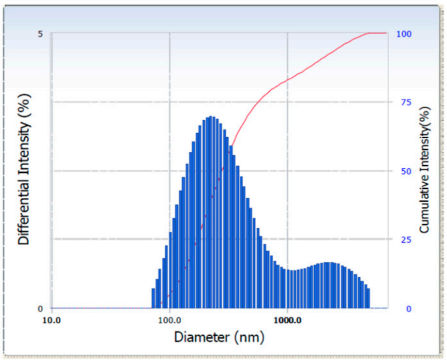

ACF

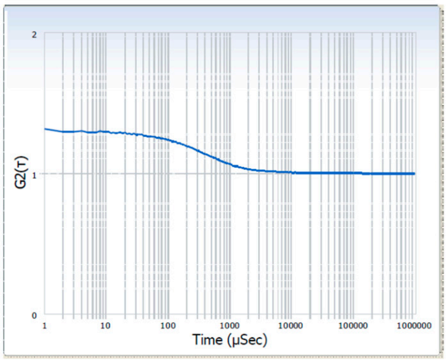

Distribution Results (Contin)

Peak	Diameter (nm)	Std. Dev.

Cumulants Results

Diameter	(d)	: 436.5	(nm)
Polydispersity Index	(P.I.)	: 0.197	

Figure S3.1(c1) Polydispersity index for AuNPsEaqSW

Mobility Distribution

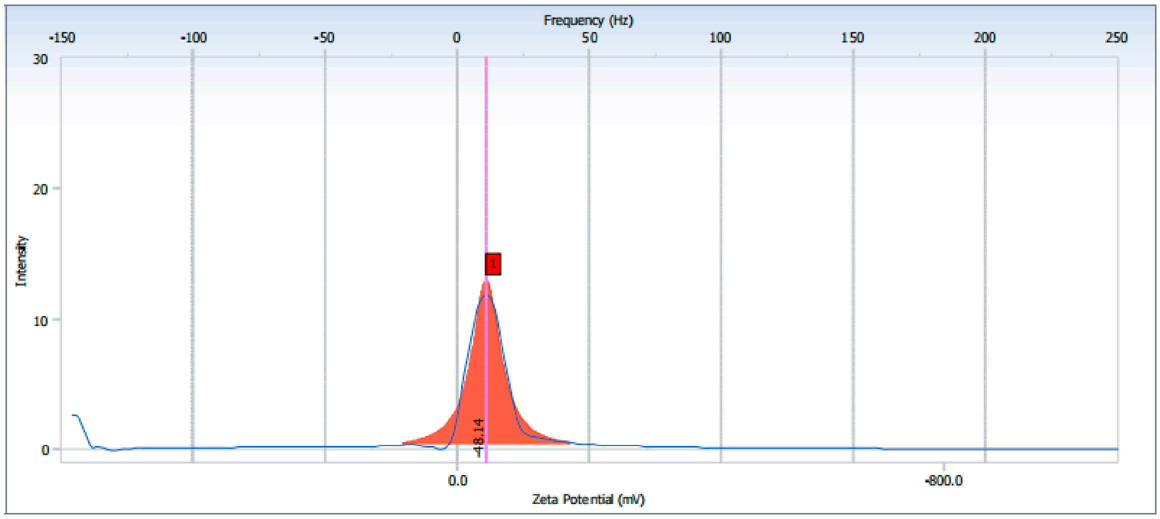

Measurement Results

Zeta Potential	: -48.14	(mV)	Doppler shift	: 11.11	(Hz)

Mobility Distribution

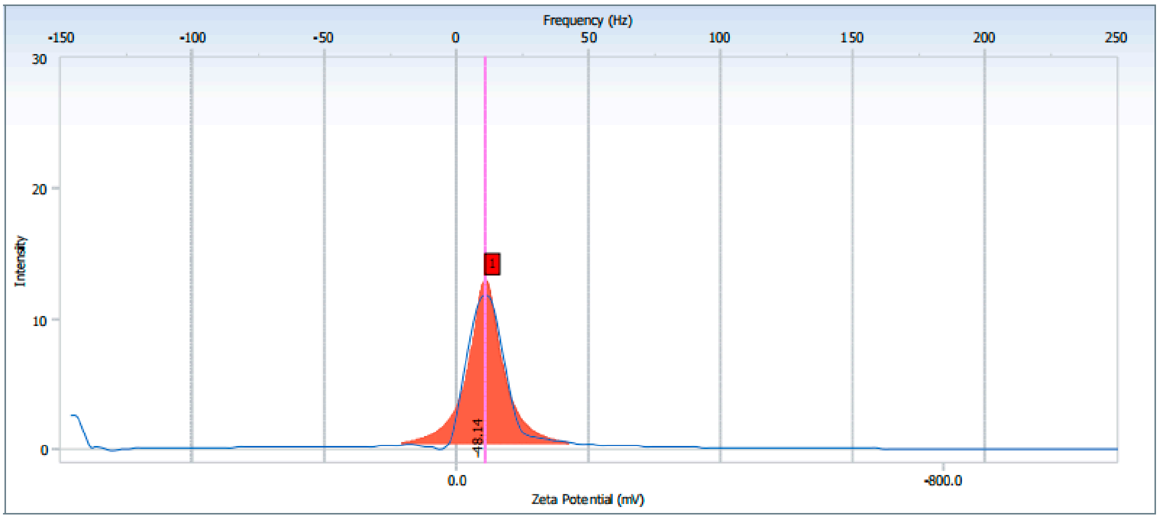

Measurement Results

Zeta Potential	: -48.14	(mV)	Doppler shift	: 11.11	(Hz)

Figure 3.1(c2) Zeta Potential for AuNPsEaqSW

Intensity Distribution	ACF

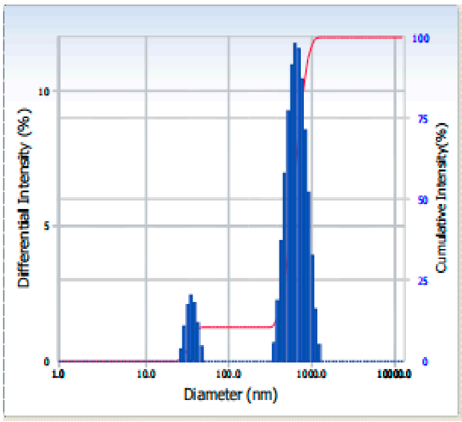

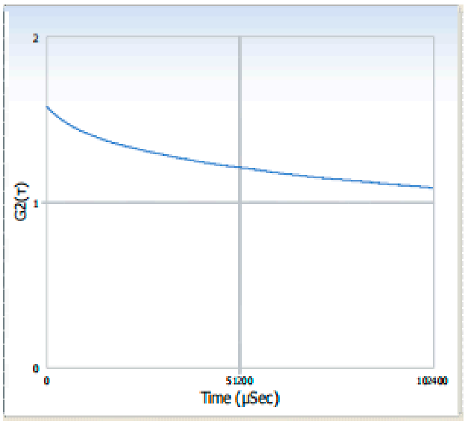

Distribution Results (Contin)			Cumulants Results			
Peak	Diameter (nm)	Std. Dev.	Diameter	(d)	: 449.9	(nm)
			Polydispersity Index (P.I.)		: 0.653	

Figure S3.1(d1) Hydrodinamic diameter, polydispersity index for AuNPsEETOHSW

Mobility Distribution

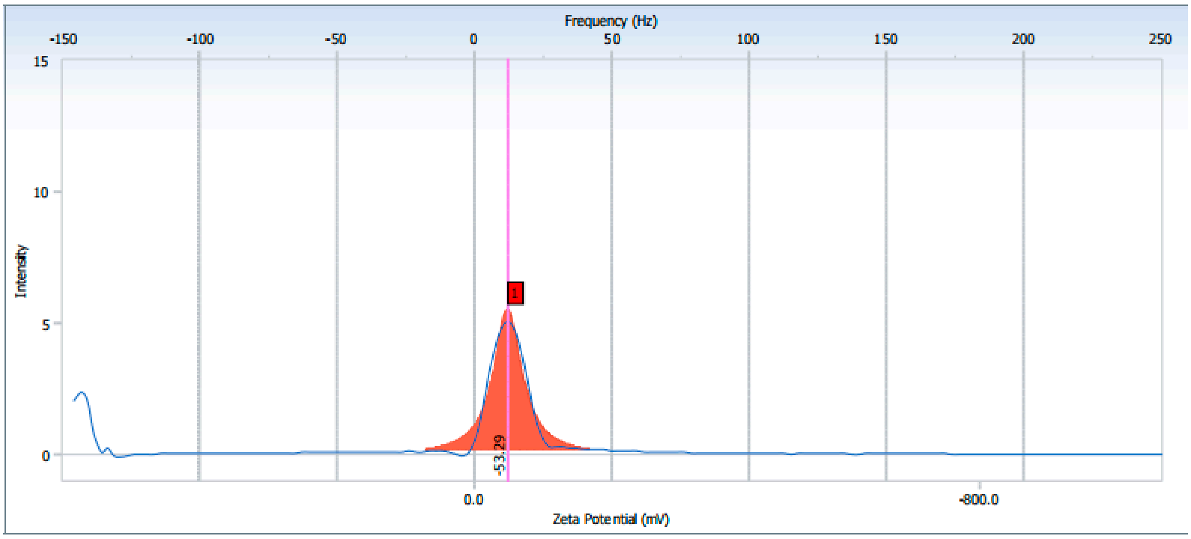

Measurement Results

Zeta Potential	: -53.29	(mV)	Doppler shift	: 12.24	(Hz)

Figure 3.1(d2) Zeta Potential for AuNPsEETOHSW

Intensity Distribution

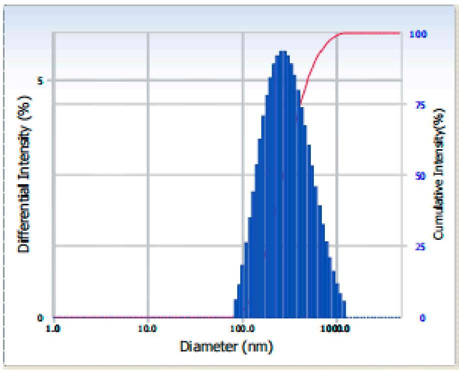

ACF

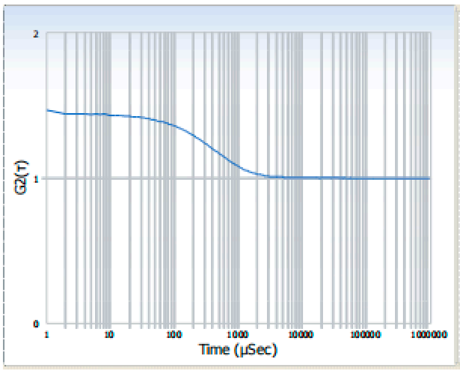

Distribution Results (Contin)

Peak	Diameter (nm)	Std. Dev.

Cumulants Results

Diameter (d) : 337.4 (nm)

Polydispersity Index (P.I.) : 0.158

Figure S3.2(a1) Hydrodinamic diameter, polydispersity index for AgNPsEaqD

Mobility Distribution

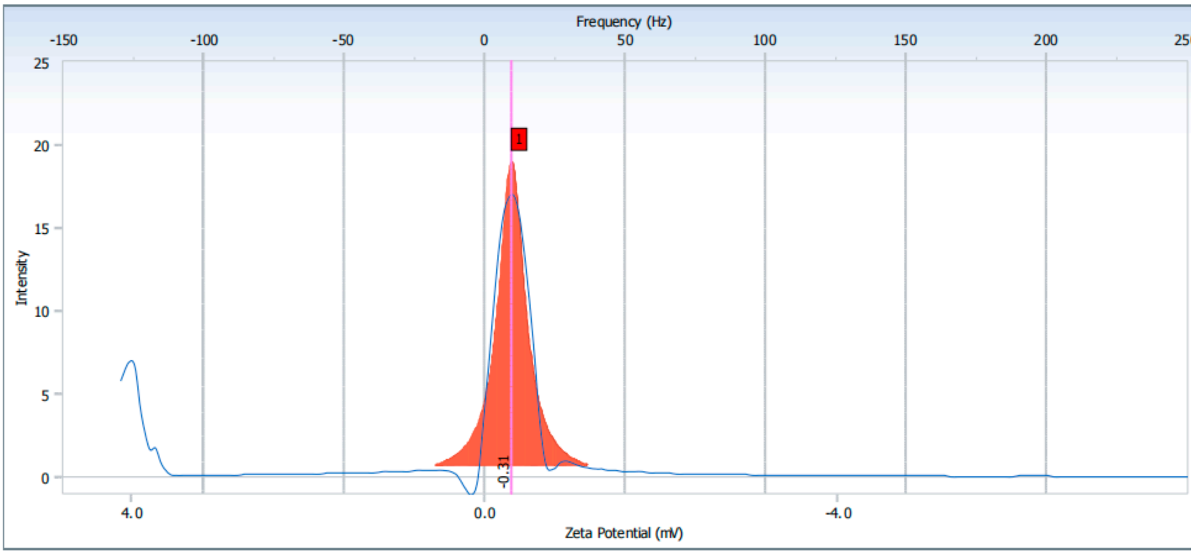

Measurement Results

Zeta Potential : -0.31 (mV) Doppler shift : 9.85 (Hz)

Figure S3.2(a2) Zeta Potential for AgNPsEaqD

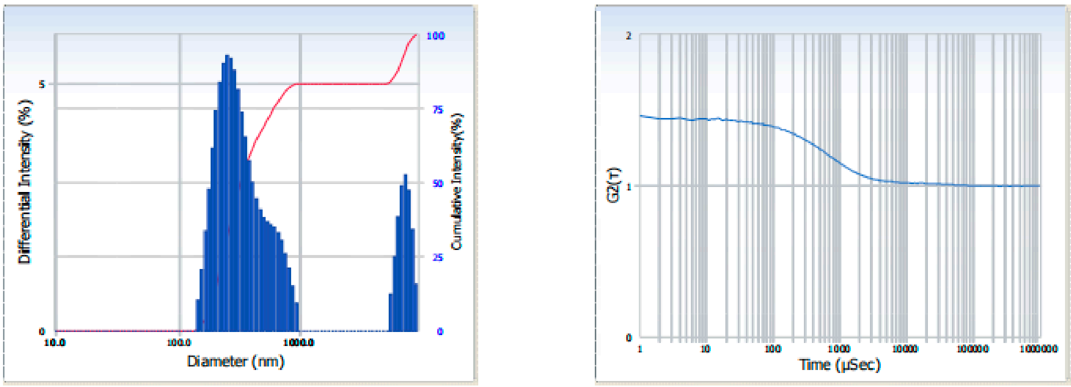

Distribution Results (Contin)			Cumulants Results	
Peak	Diameter (nm)	Std. Dev.	Diameter (d)	: 510.6 (nm
			Polydispersity Index (P.I.)	: 0.258

Figure S3.2(b1) Hydrodinamic diameter, polydispersity index for AgNPsEtOHD

Mobility Distribution

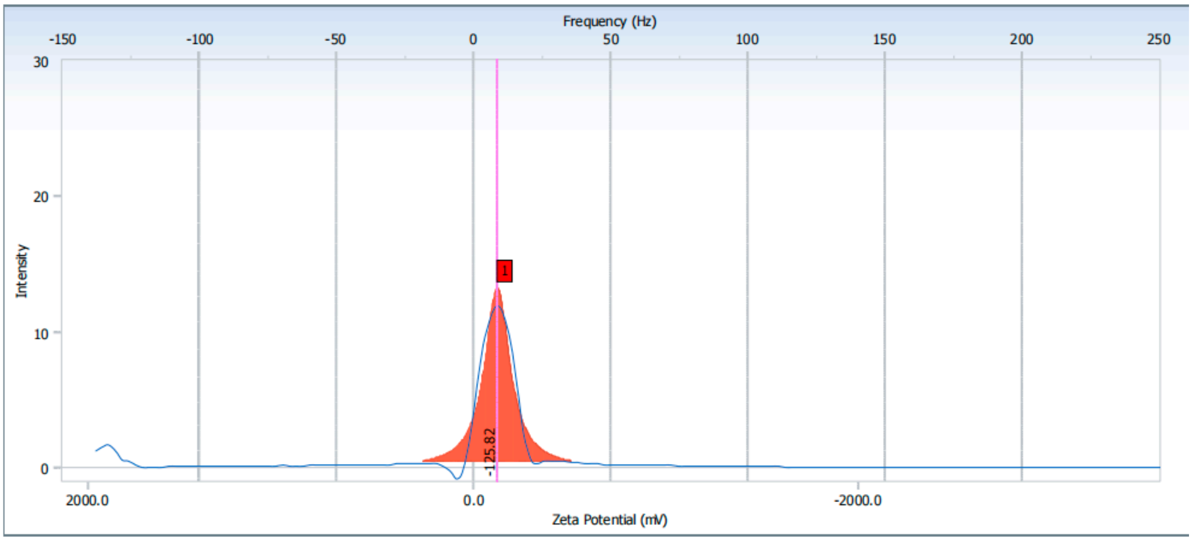

Measurement Results

Zeta Potential	: -125.82	(mV)	Doppler shift	: 8.82	(Hz)

Figure S3.2(b2) Zeta Potential for AgNPsEtOHD

Intensity Distribution

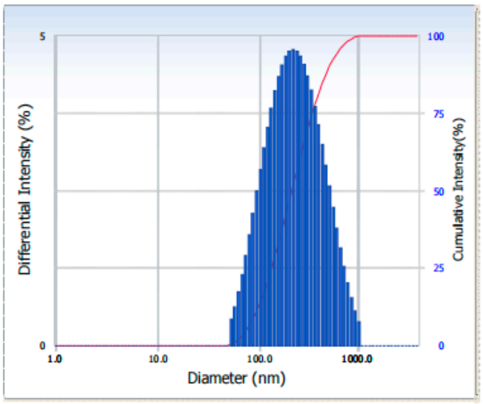

ACF

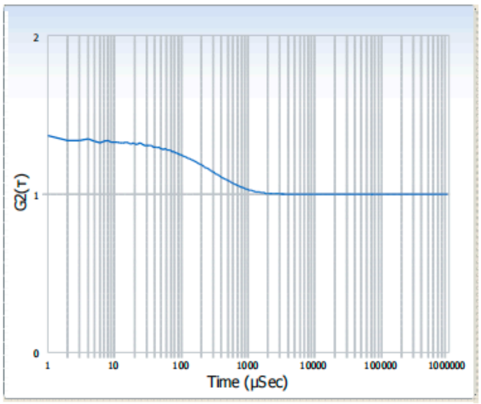

Distribution Results (Contin)

Peak	Diameter (nm)	Std. Dev.

Cumulants Results

Diameter (d) : 202.8 (nm

Polydispersity Index (P.I.) : 0.264

Figure S3.2(c1) Hydrodinamic diameter, polydispersity index for AuNPsEaqD

Mobility Distribution

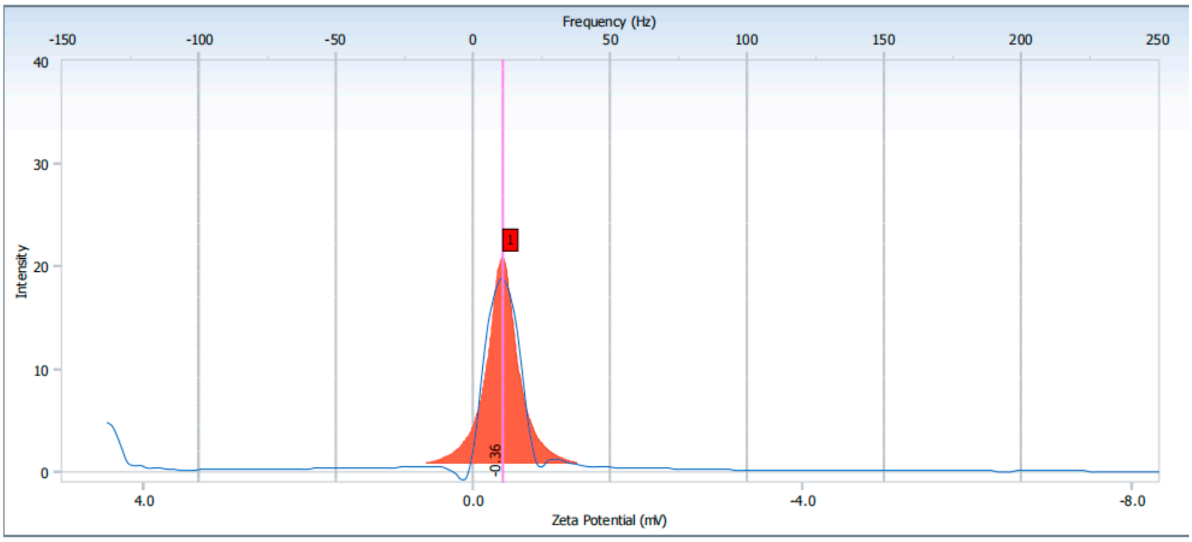

Measurement Results

Zeta Potential : -0.36 (mV) Doppler shift : 10.84 (Hz)

Figure S3.2(c2) Zeta Potential for AuNPsEaqD

Intensity Distribution

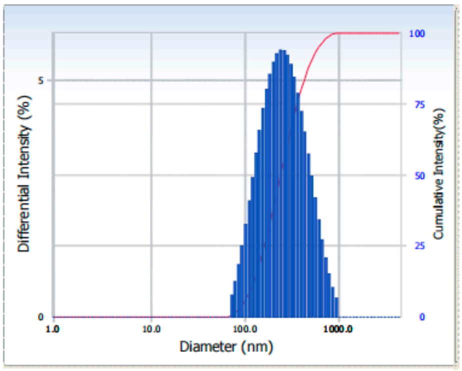

ACF

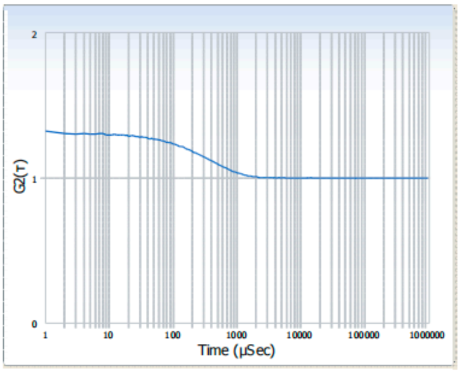

Distribution Results (Contin)

Peak	Diameter (nm)	Std. Dev.

Cumulants Results

Diameter	(d)	: 245.6	(nm)
Polydispersity Index	(P.I.)	: 0.260	

Figure S3.2(d1) Hydrodinamic diameter, polydispersity index for AuNPsE$_{ETOH}$D

Mobility Distribution

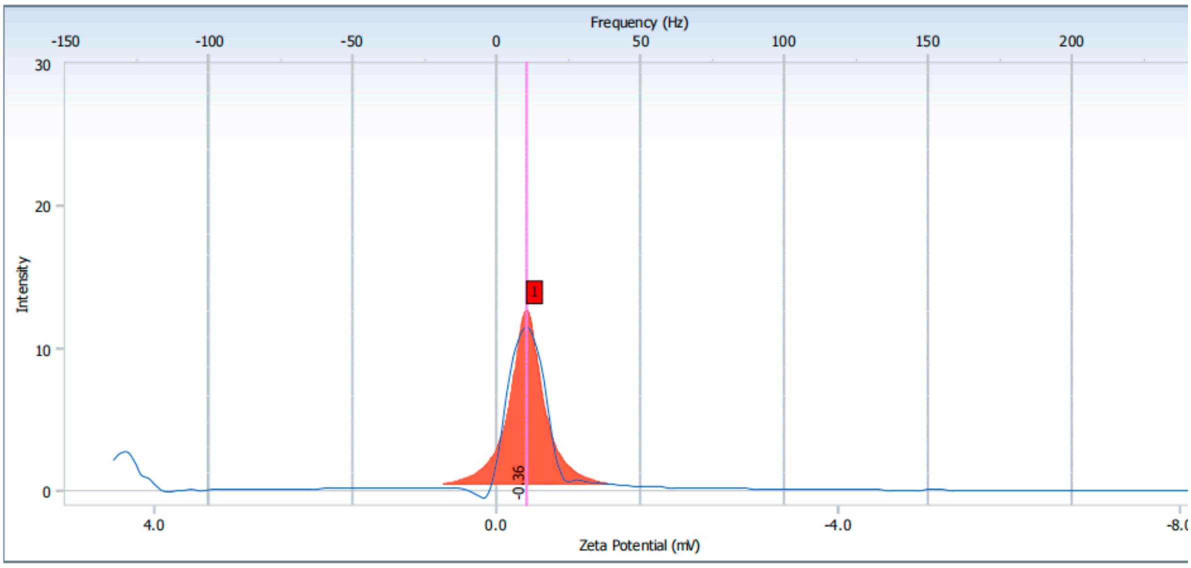

Measurement Results

Zeta Potential	: -0.36	(mV)	Doppler shift	: 10.58	(Hz)

Figure S3.2(d2) Zeta Potential for AuNPsE$_{ETOH}$D

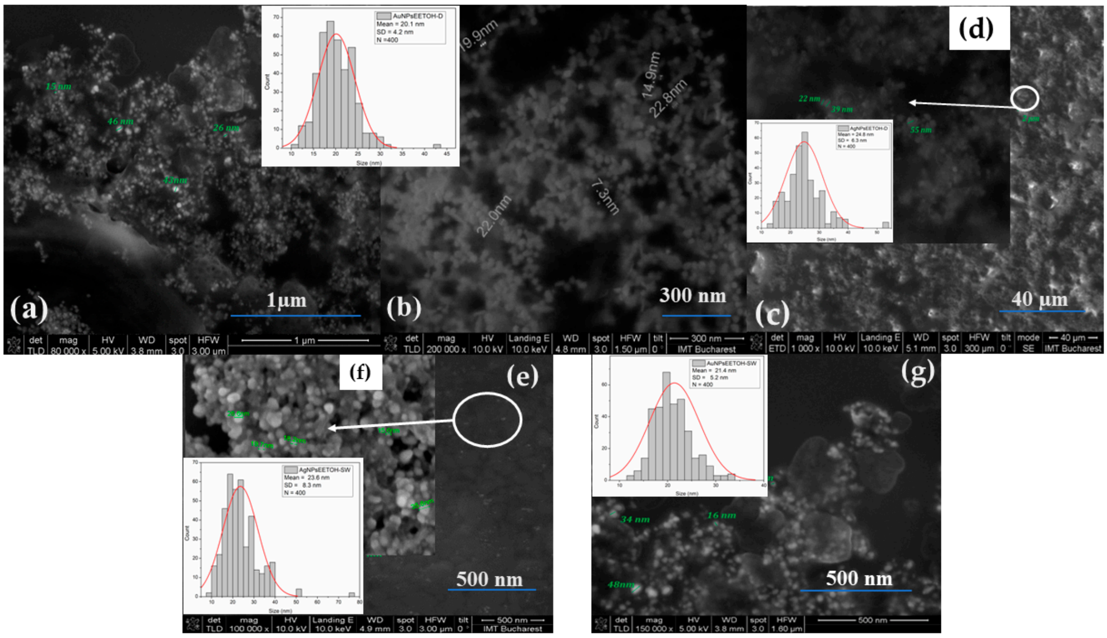

Figure S4. SEM images of at different magnitudes for **a.** AuNPsE$_{ETOH}$-D (80 000x); **b.** AuNPsE$_{ETOH}$-D (200 000x); **c.** AgNPsE$_{ETOH}$-D (1000 x); **d.** AgNPsE$_{ETOH}$-D (200 000 x); **e.** AgNPsE$_{ETOH}$-SW (100 000x); **f.** AgNPsE$_{ETOH}$-SW (300 000x); g. AuNPsE$_{ETOH}$-SW (150 000x).

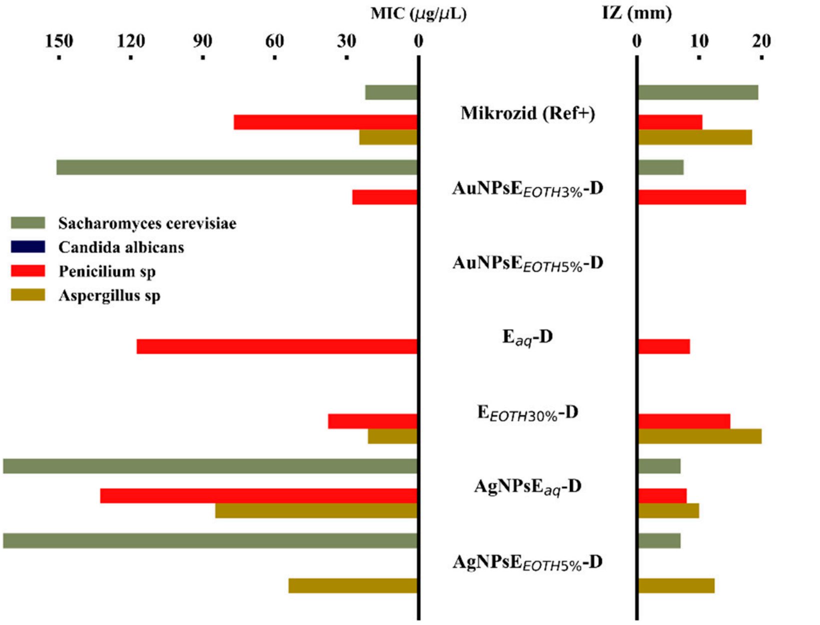

Figure S5. Antifungal effect of Eaq/$_{ETOH}$ and for nanoparticles from Dandelion

Legend: Mikrozid – alchoolic solution 60%; E$_{ETOH50\%}$-D – alchoolic extracts 50% from Dandelion; E$_{ETOH30\%}$-D- achoolic extract 30% from Dandelion; AgNPsE$_{ETOH5\%}$- Dandelion- obtained by silver reduction with E$_{ETOH50\%}$-D (1 :10, extract: AgNO3 1mM – v:v); AuNPsE$_{ETOH3\%}$-D obtained by gold reduction with E$_{ETOH30\%}$-D (1 :10, extract: HAuCl4 0.5mM – v:v); AuNPsE$_{ETOH5\%}$-D obtained by gold reduction with E$_{ETOH50\%}$-D (1 :10, extract: HAuCl4 0.5mM – v:v)

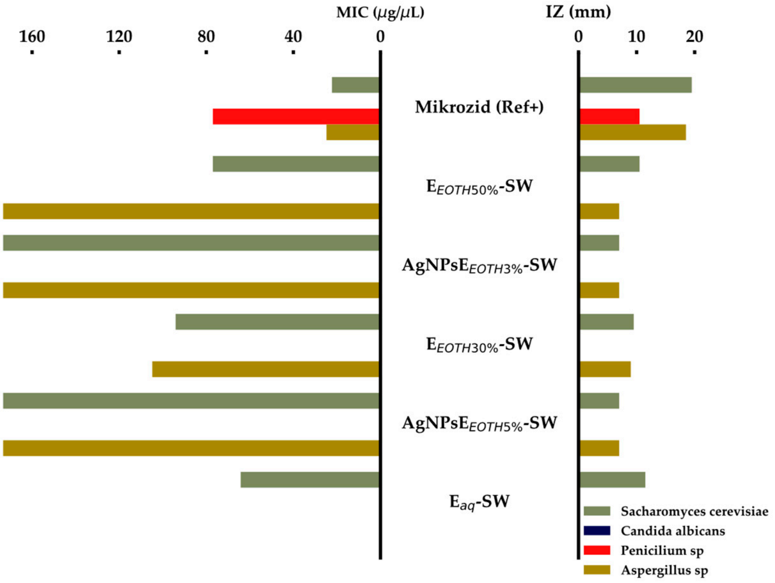

Figure S6. Antifungal effect of Eaq/$_{ETOH}$ and for nanoparticles from Sweet Wormood

Legend: Mikrozid – alchoolic solution 60%; E$_{ETOH50\%}$-SW – alchoolic extracts 50% from Sweet Wormood; E$_{ETOH30\%}$-SW- achoolic extract 30% from sweet wormood; AgNPsE$_{ETOH5\%}$- SW- obtained by silver reduction with E$_{ETOH50\%}$-SW (1 :10, extract: AgNO3 1mM – v:v); AgNPsE$_{ETOH3\%}$- SW- obtained by silver reduction with E$_{ETOH30\%}$-SW (1 :10, extract: AgNO3 1mM – v:v)

Table S2. Antibacterial effect of Eaq/ETOHD at 100 µL diffusimetric ABG (IZ and MIC)

No	Substance name	Measured/calculated effect				Remarks/ rank effect
		S.a.	*E.c.*	*P.a.*	*B.s.*	
		IZ(mm)/ MIC(µg/ µL)	IZ(mm)/ MIC(µg/µL)	IZ(mm)/ MIC(µg/µL)	IZ(mm)/ MIC(µg/µL)	
1	AgNPsEETOH5%-D	17,5/138, 7	30,0/ 47,2	20,0/106,2	0,0/-	Synergic 3 1
2	AgNPsEaq-D	13,0/251, 3	10,0/424,6	13,5/233 (RM)	0,0/-	RM*, 3
3	EETOH 3%-D	13,5/233	20,0/106,2	0,0/ -	0,0/ -	Synergic 1 4
4	EaqD	0,0/-	0,0/ -	0,0/-	0,0/ -	6
5	AuNPsEETOH 5%-D	9,0/524,1	20,0/106,2 (RM)	0,0/-	0,0/ -	RM*, 5
6	AuNPsEETOH 3%-D	25,0/ 67,9	10,5/ 385,2	10,0/ 424,6	0,0/ -	2
7	$HAuCl_4$ X3H_2O 0.5mM	0	0	0	0	0
8	AgNO3 1mM	0	0	0	0	0
	Registered RM cases	0	1	1	0	2
	Remarks / sensitivity rank	2	1	3	4	

Legend: Ag/AuNPsE$_{ETOH3\%\ sau\ 5\%}$D: Dandelion extract with Ag/Au NPs in Ethanol 3% sau 5%.Ag/AuNPsEaqD: Dandelion extract with Ag/AuNPs in water.

S.a.: Staphylococcus aureus; E.c.: Escherichia coli; P.a.: Pseudomonas aeruginosa, B.s.: Bacillus subtilis.

IZ (mm): the diameter of the inhibiton zone in mm, MIC(µg/µL): minimum inhibitory concentration in µg/µL.

*RM = resistant mutant

Blank Solutions – 0.5 mM HAuCl4x3H20 and 1mM AgNO3 solution have no antimicrobial effect.

Table S3. Antibacterial effect of Eaq/ETOHD at 20 μL diffusimetric ABG (IZ and MIC)

No	Substance name	Measured/calculated effect				Remarks/ rank effect
		S.a.	*E.c.*	*P.a.*	*B.s.*	
		IZ(mm)/ MIC(μg/μL)	IZ(mm)/ MIC(μg/μL)	IZ(mm)/ MIC(μg/μL)	IZ(mm)/ MIC(μg/μL)	
1	AgNPsEETOH5%D	9,0/104,8	0,0/-	0,0/-	7,0/173,3	3
2	AgNPsEaqD	7,0/173,3	0,0/-	0,0/-	7,0/173,3	4
3	EETOH 3%D	9,5/94,1	0,0/-	0,0/-	9,0/104,8	1
4	EaqD	0,0/-	0,0/-	0,0/-	7,0/173,3	5
5	AuNPsEETOH 3%D	7,0/173,3	0,0/-	0,0/-	7,0/173,3	4
6	EETOH 3%D	10,5/77,0	0,0/-	0,0/-	7,0/173,3	2
	Registered RM cases	0	0	0	0	0
	Remarks / sensitivity rank	2	-	-	1	

Blank Solutions – 0.5 mM HAuCl4x3H20 and 1mM AgNO3 solution have no antimicrobial effect.

Table S4. Antifungal effect of Eaq/ETOHD at 20 μL diffusimetric AFG (IZ and MIC)

Legend: *S.c.*: *Saccharomyces cerevisiae, C.a.: Candida albicans, Pen.: Penicillium sp.: Asp.: Aspergillus sp.*

No	Substance name	Measured/calculated effect				Remarks/ rank effect
		S.c.	*C.a.*	*Pen.*	*Asp.*	
		IZ(mm)/ MIC(μg/μL)	IZ(mm)/ MIC(μg/μL)	IZ(mm)/ MIC(μg/μL)	IZ(mm)/ MIC(μg/μL)	
1	AgNPsEETOH5%-D	7,0/173,3	0,0/-	0,0/-	12,5/54,3 (RM)	RM, 3
2	AgNPsEaq-D	7,0/173,3	0,0/-	8,0/132,7	10,0/84,9 (RM)	RM, 2
3	EETOH 3%-D	0,0/-	0,0/-	15,0/37,7	20,0/21,2	1
4	EaqD	0,0/-	0,0/-	8,5/117,6 (RM)	0,0/-	RM, 4
5	AuNPsEETOH 3%-D	0,0/-	0,0/-	0,0/-	0,0/-	5
6	EETOH 3%-D	7,5/150,9	0,0/-	17,5/27,7	0,0/-	2
	Registered RM cases	0	0	1	2	3
	Remarks / sensitivity rank	3	4	1	2	

Blank Solutions – 0.5 mM HAuCl4x3H20 and 1mM AgNO3 solution have no antimicrobial effect.

Table S5. Antibacterial effect of Eaq/ETOHSW extracts at diffusimetric ABG (IZ and MIC)

No	Substance name	Measured/calculated effect				Remarks/ rank effect
		S.a.	*E.c.*	*P.a.*	*B.s.*	
		IZ(mm)/ MIC(μg/μL)	IZ(mm)/ MIC(μg/μL)	IZ(mm)/ MIC(μg/μL)	IZ(mm)/ MIC(μg/μL)	
1	Eaq-SW/100 μL	8,0/663,6	22,5/83,9	30,0/47,2	20,0/106,2	1
2	AgNPsEETOH5%SW/100 μL	27,5/56,1	13,5/233,0	27,0/58,2 (RM)	0,0/-	RM, 2
3	AgNPsEETOH5%SW/100 μL	15,0/188,7	10,0/424,6	9,5/470,6	0,0/-	3

4	Eaq-SW/20 µL	11,5/64,2	0,0/-	0,0/-	0,0/-	5
5	AgNPsEaq-SW/20 µL	11,0/70,2	11,0/70,2	8,5/117,6	11,5/64,2	4
	Registered RM cases	0	0	1	0	1
	Remarks / sensitivity rank	3	2	1	4	

Legend: Ag/AuNPsEETOH3% sau 5%SW: SW extract with Ag/Au NPs in Ethanol 3% sau 5%, Ag/AuNPsEaqSW: SW extract with Ag/AuNPs in water.

Blank Solutions – 0.5 mM HAuCl4x3H20 and 1mM AgNO3 solution have no antimicrobial effect.

Table S6. Antifungal effect of Eaq/ETOHDW extracts at diffusimetric AFG with 20 µL (IZ and MIC)

No	Substance name	Measured/calculated effect				Remarks/ rank effect
		S.c.	*C.a.*	*Pen.*	*Asp.*	
		**IZ(mm)/ MIC(µg/µL)**	**IZ(mm)/ MIC(µg/µL)**	**IZ(mm)/ MIC(µg/µL)**	**IZ(mm)/ MIC(µg/µL)**	
1	EaqSW	11,5/64,2	0,0/-	0,0/-	0,0/-	3
2	AgNPsEaq-SW	7,0/173,3	0,0/-	0,0/-	7,0/173,3	4
3	EETOH3%-SW	9,5/94,1	0,0/-	0,0/-	9,0/104,8	1
4	EaqSW	0,0/-	0,0/-	0,0/-	7,0/173,3	5
5	AuNPsEETOH 3%-SW	7,0/173,3	0,0/-	0,0/-	7,0/173,3	4
6	EETOH 3%-SW	10,5/77,0	0,0/-	0,0/-	7,0/173,3	2
	Registered RM cases	0	0	0	0	0
	Remarks / sensitivity rank	2	-	-	1	

Blank Solutions – 0.5 mM HAuCl4x3H20 and 1mM AgNO3 solution have no antimicrobial effect.

Table S7. Comparative cytotoxic effects of the tested formulations on different cell lines in relation to Cisplatin (CisPt) and Doxorubicin (DOX).

Comparision	Effect*	p-value
**LoVo Group ($\chi 2$ = 58.428; p = 2.56e-11)**		
**EETOH30%-SW vs Cis-Pt**	-	2.28e-4
**AgNPsEETOH3%-D vs Cis-Pt**	+	4.58e-3
**AuNPsEaq-D vs Cis-Pt**	-	1.82e-3
**MDA-MB ($\chi 2$ = 42.035; p = 5.79e-8)**		
**EETOH30%-SW vs DOX**	+	4.58e-3
**AgNPsEETOH3%-SW vs DOX**	+	1.43e-8
**AgNPsEETOH3%-D vs DOX**	+	7.20e-5
**AuNPsEETOH3%-D vs DOX**	+	4.72e-4
**HUVEC ($\chi 2$ = 82.848; p = 9.21e-16)**		
**AgNPsEETOH3%-D vs Cis-Pt**	+	1.87e-3
**AuNPsEaq-D vs Cis-Pt**	-	1.44e-5
**AuNPsEETOH3%-D vs Cis-Pt**	-	7.93e-4
**EETOH30%-SW vs DOX**	-	1.06e-3
**AuNPsEaq-D vs DOX**	-	1.01e-8
**AuNPsEETOH3%-D vs DOX**	-	2.07e-6
**HepG2 ($\chi 2$ = 105.868; p = 1.67e-19)**		
**AgNPsEETOH5%-SW vs Cis-Pt**	-	1.40e-4
**AuNPsEETOH5%-SW vs Cis-Pt**	-	9.19e-9
**AuNPsEaq-SW vs Cis-Pt**	-	1.40e-4
**EEOTH30%-D vs CisPt**	-	1.59e-9
**AgNPsEETOH5%-D vs Cis-Pt**	-	7.00e-9
**AgNPsEaq-D vs Cis-Pt**	-	3.49e-9

*The effect symbol ('+') indicates that the tested compound demonstrated higher cytotoxicity compared to the control, whereas ('-') signifies higher cell viability, indicating a weaker cytotoxic effect. The statistical significance of each comparison is represented by the corresponding p-values.

Table S8. IC50, SI (and their corresponding errors) at 24 h

	Compound	IC50 Value			IC50 Error			Selectivity Index	
1		HUVEC	LoVo	MDA-MB	HUVEC	LoVo	MDA-MB	LoVo	MDA-MB
3	E(ETOH30%) - SW	0,40557	0,82629	0,06848	0,21759	1,52709	0,00833	0,49084	5,92291
4	AgNPsE(ETOH3%) - SW	0,10875	0,07544	0,04909	0,01550	0,00926	0,03517	1,44147	2,21517
5	AgNPsE(ETOH3%) - D	0,06028	0,05276	0,04544	0,00491	0,01015	0,00386	1,14258	1,32641
6	AuNPsE(aq) - D	0,06818	0,17986	0,21951	0,02288	0,02200	0,16547	0,37905	0,31059
7	AuNPsE(ETOH3%) - D	0,07299	0,22175	0,45575	0,02183	0,02862	0,87651	0,32916	0,16016
8	DOX	0,05799	-	0,21982	0,00657	-	0,41662	0,00000	0,26380
9	Cis-Pt	0,05860	0,40161	-	0,04856	0,63644	-	0,14592	0,00000

Table S9. IC50, SI (and their corresponding errors) at 48 h

	Compound	IC50 Value			IC50 Error			Selectivity Index	
1		HUVEC	LoVo	MDA-MB	HUVEC	LoVo	MDA-MB	LoVo	MDA-MB
3	E(EOTH30%) - SW	0,18120	0,10972	0,02412	0,21784	0,01601	0,04639	1,65144	7,51239
4	AgNPsE(EOTH3%) - SW	0,08154	0,05634	0,21758	0,01271	0,01918	0,07088	1,44733	0,37475
5	AgNPsE(EOTH3%) - D	0,04419	0,05005	0,01724	0,01181	0,01364	0,02048	0,88290	2,56244
6	AuNPsE(aq) - D	0,23576	0,10895	0,02800	0,06323	0,01232	0,04892	2,16387	8,41974
7	AuNPsE(EOTH3%) - D	0,13605	0,23898	0,36692	0,04482	0,28763	0,54833	0,56929	0,37079
8	DOX	0,05447	-	0,09422	0,00598	-	0,04560	0,00000	0,57814
9	Cis-Pt	0,05015	0,38897	-	0,01745	0,30715	-	0,12893	0,00000
10									

Table S10. IC50, SI (and their corresponding errors) at 24 h and 48h after HepG2 treatments

	Compounds	IC50 Value		IC50 Error		Selectivity Index	
1		24 h	48 h	24 h	48 h	24 h	48 h
3	E(EOTH30%) - SW	0,30306	0,22284	0,18159	0,10030	1,33826	0,81314
4	AgNPsE(EOTH5%) - SW	0,18025	0,27667	0,07784	0,41994	0,60332	0,29471
5	AgNPsE(aq) - SW	0,06040	0,34611	0,00838	0,34678	0,00000	0,00000
6	AuNPsE(EOTH5%) - SW	0,18850	0,26723	0,10508	0,20916	0,00000	0,00000
7	AuNPsE(aq) - SW	0,16503	0,21650	0,05262	0,21391	0,00000	0,00000
8	E(EOTH30%) - D	0,06983	0,38912	0,00731	0,54871	0,00000	0,00000
9	AgNPsE(EOTH5%) - D	0,10202	0,12298	0,01466	0,17821	0,59087	0,35930
10	AgNPsE(aq) - D	0,03920	0,16191	0,02966	0,05458	0,00000	0,00000
11	Cis - Pt	0,38118	0,32404	0,58638	0,21436	0,15374	0,15477
